# Detection of Single Charge Trapping Defects in Semiconductor Particles by Evaluating Photon Antibunching in Delayed Photoluminescence

**DOI:** 10.1021/acs.nanolett.2c04004

**Published:** 2023-03-09

**Authors:** Ivan Yu. Eremchev, Aleksandr O. Tarasevich, Maria A. Kniazeva, Jun Li, Andrei V. Naumov, Ivan G. Scheblykin

**Affiliations:** † Institute of Spectroscopy RAS, Troitsk, Moscow 108840, Russia; ‡ Lebedev Physical Institute of the Russian Academy of Sciences, Troitsk, Moscow 108840, Russia; § Chemical Physics and Nano Lund, Lund University, Box 124, SE-22100 Lund, Sweden; ∥ National Research University Higher School of Economics, Moscow 109028, Russia

**Keywords:** single trap detection, antibunching, delayed photoluminescence, single perovskite submicron crystals, defect, Auger recombination

## Abstract

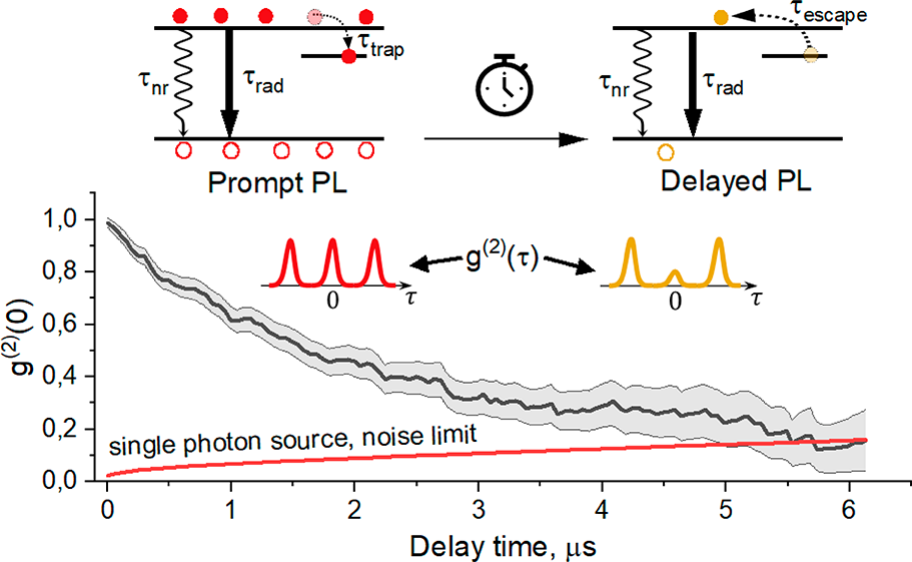

Time-resolved analysis of photon cross-correlation function *g*^(2)^(τ) is applied to photoluminescence (PL) of individual submicrometer size MAPbI_3_ perovskite crystals. Surprisingly, an antibunching effect in the long-living tail of PL is observed, while the prompt PL obeys the photon statistics typical for a classical emitter. We propose that antibunched photons from the PL decay tail originate from radiative recombination of detrapped charge carriers which were initially captured by a very limited number (down to one) of shallow defect states. The concentration of these trapping sites is estimated to be in the range 10^13^–10^16^ cm^–3^. In principle, photon correlations can be also caused by highly nonlinear Auger recombination processes; however, in our case it requires unrealistically large Auger recombination coefficients. The potential of the time-resolved *g*^(2)^(0) for unambiguous identification of charge rerecombination processes in semiconductors considering the actual number of charge carries and defects states per particle is demonstrated.

Antibunching is a phenomenon intrinsic to luminescence photons emitted by single emitters like single atoms^1,2^ and molecules.^3,4^ The effect is due to the inability of a single two-level system to emit two photons simultaneously. Photon antibunching is not only inherent to classical two-level systems, but also common for a wide range of nanoemitters including semiconductor quantum dots (QDs), perovskite nanocrystals (NCs),^5−7^ π-conjugated polymers,^8,9^ and light-harvesting complexes.^10^ Observation of the antibunching effect in extended systems like polymers and aggregates and studying of their emission photon statistics allowed for a deeper insight into photophysical processes at the level of individual chromophores and the nature of their excited states.^11−13^ This also applies to inorganic emitters where photon statistics combined with spectroscopy allowed for understanding of the multiexcitonic states and interactions between them.^14−16^ The general photon statistics of a nanoemitter can be nontrivial and time-dependent because the charge recombination mechanism can depend strongly not only on the number of charge carriers present at the particular moment of time but also on the whole evolution history of the excited state population started by the laser pulse. Thus, measuring photon correlations as a function of time appears as a powerful tool for rationalizing fundamental photophysical properties of luminescent materials.^13^

Metal halide perovskites (MHPs)^17^ are solution-processed semiconductors with very interesting photophysics and highly suitable properties for optoelectronic applications. Today MHPs are available with almost any bandgap energy from UV to NIR and in the form ranging from nano- and microcrystals to polycrystalline films and single crystals with sizes up to several millimeters and larger.^18−20^

MHPs are very strong light absorbing and highly luminescent materials, where the evolution of the photoexcited charge carriers is affected by different types of defects not only introduced during synthesis, but also appearing later under the influence of light, electric field, or current.^19,21−23^ The remarkable peculiarity of MHPs is that formation of defects and degradation of the material performance very often are fully reversible due to defect metastability making MHPs able to self-heal.^24,25^

MHP crystals with sizes below one micrometer may contain just several efficient nonradiative (NR) recombination centers.^26^ Concentrations of NR centers measured by spectroscopy methods are commonly of the order of 10^15^ cm^–3^ corresponding to one NR center per cube with a 100 nm side.^27^ A small number of metastable NR centers per an individual object leads to a photoluminescence (PL) blinking effect observed for MHP crystals of various sizes.^28−30^ Efficient charge carrier migration over hundreds of nanometers allows electrons and holes to reach the NR recombination center. In this case activating/deactivating of just one metastable NR center (often called “supertrap”^30^) leads to large jumps of the NR recombination rate appearing as PL blinking. This blinking mechanism originally proposed for conjugated polymers^31^ is also known as the model of multiple recombination centers^32^ for QDs, and in the field of perovskites it is referred as the supertrap model.^28,30^

The supertrap model has been recently supported by photon correlation experiments.^33^ It was demonstrated that the PL of an individual MAPbI_3_ submicrometer crystal does not exhibit photon antibunching despite possessing a pronounced PL blinking. This demonstrates that there is no mechanism, which would suppress simultaneous emission of two photons from such large crystals. Indeed, the size of the crystals is too large to result in Auger recombination of photoexcited charge carriers in a charged crystal which is responsible for suppressing multiphoton emission.^33^

Many MHP systems show a microsecond-long tail in the time-resolved PL decay.^34−38^ Nonexponentiality of the PL decay is often observed for a material with free charge carriers exhibiting concentration-dependent Auger and radiative recombination rates. However, the tail also has a contribution from so-called delayed PL occurring from radiative recombination of initially trapped charge carriers that are released again to the band. Such photons appear predominantly much later than the prompt PL photons generated by direct recombination of charge carriers before their trapping.^34^

In the current contribution, we investigate photon statistics of the delayed PL in single submicrometer methylammonium lead tri-iodide (MAPbI_3_) perovskite crystals. In agreement with our previous result,^33^ the antibunching effect is not observed in a time-integrated PL signal. However, surprisingly, a partial antibunching appears when the delayed component of the PL decay is analyzed separately. In many cases the extent of antibunching is larger when the PL become partially quenched during PL blinking. We propose that this partial antibunching means that the studied crystals contained a very small number of charge trapping states responsible for the delayed PL. Our results show that measuring the photon correlation function as a function of time is a very sensitive method for detecting long-living states and revealing electronic processes at extremely low concentration of excitations in luminescent materials.

We studied photon statistics of individual MAPbI_3_ crystals with a characteristic size from tens of nanometers to a few micrometers using a home-built luminescence microscope setup equipped with the Hanbury Brown and Twiss correlation scheme (Supporting Information (SI) Supporting Note 1 (SN1)). The average number of excitations per laser pulse per crystal (for the crystals appearing as diffraction-limited spots in PL images) is estimated to be from 7.5 to 70 (SI SN9). The second order cross-correlation function *g*^(2)^(τ), where τ is the time shift between two photons, was calculated for the photons arriving to the detector after a delay *T*_D_ relative to the laser pulse. Hereafter we call this function *g*^(2)^(τ, *T*_D_). The usually reported *g*^(2)^(τ) calculated for all photons is then equal to *g*^(2)^(τ, *T*_D_ = 0). The calculation procedure is known^15,39^ and described in SI SN5. The same experimental data were also used to calculate PL decay kinetics and PL intensity traces for each crystal (SI SN4).

Figure 1 shows PL decay (panel (a)) and the normalized (see SI SN5) *g*^(2)^(τ, *T*_D_) for crystal #7 for *T*_D_ = 0 (panel (b), all photons were taken), for *T*_D_ = 4 μs (panel (c), photons from the interval 4–10 μs were taken), and for *T*_D_ = 8 μs (panel (c), photons from the interval 8–10 μs were taken). As one can see, the central peak is getting lower relative to the side peaks with an increase of *T*_D_. While there is no sign of photon antibunching when all photons are taken together (Figure 1(b)), appearance of a partial antibunching for photons belonging to the tail of the PL decay is apparent (Figure 1(c, d)). *g*^(2)^(0, *T*_D_) appears as a monotonically decaying function (Figure 1(e)) changing from 1 for all photons (expected for the Poisson statistics) to 0.6 for the photons arriving after 8 μs from the excitation pulse. It means that the photon statistics of the delayed PL of this microcrystal approaches that of a single photon source.

**Figure 1:**
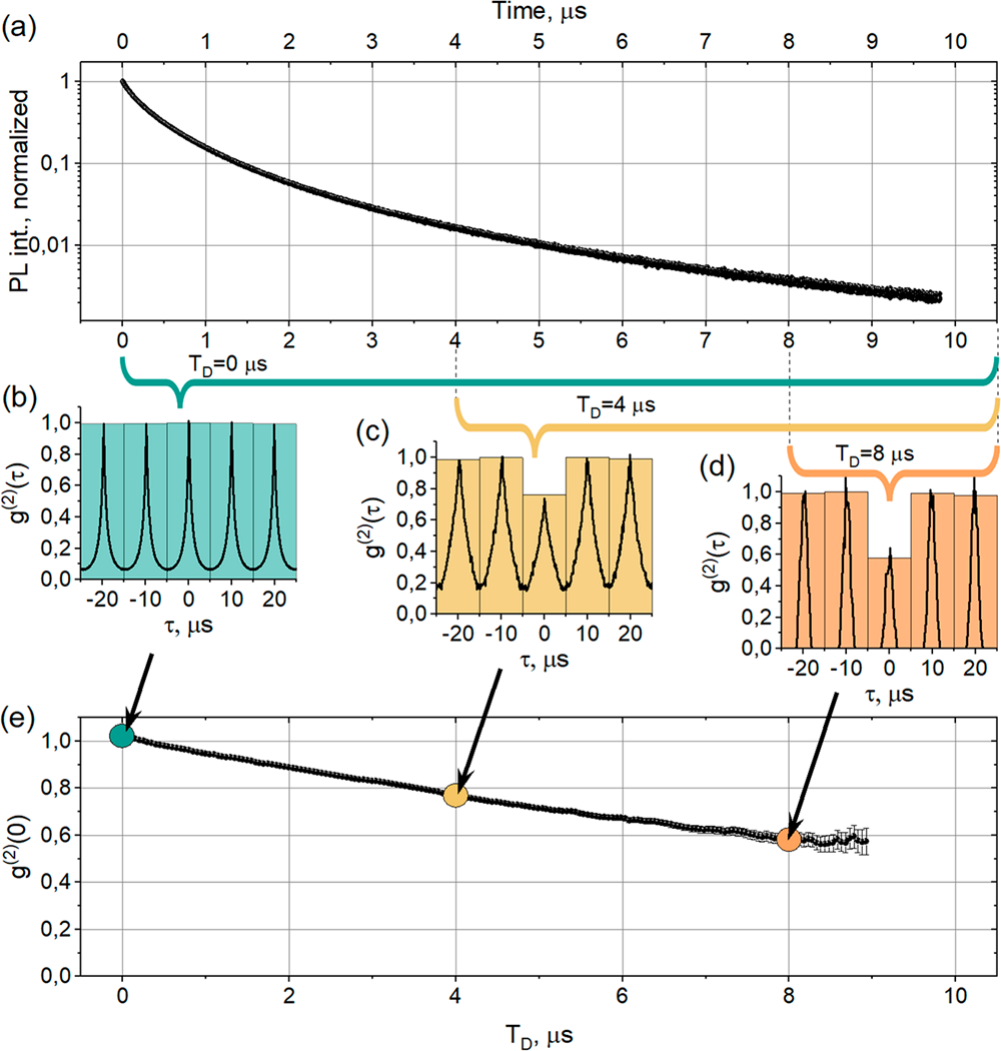
Evolution of the second order cross-correlation function *g*^(2)^(τ) measured for an individual MAPbI_3_ submicrometer crystal #7 as a function of the time delay (*T*_D_) passed from the arrival of the excitation pulse. (a) PL decay kinetics showing a pronounced tail at long times. Three time intervals starting at time delay *T*_D_ = 0, 4, and 8 μs and ending at *T*_max_ = 10 μs (arrival time of the next laser pulse) are marked with green, dark yellow, and orange colors. Photons from these time intervals are used to calculate normalized *g*^(2)^(τ, *T*_D_) shown in (b), (c), and (d). The height of the bars is the integral over each peak of *g*^(2)^(τ, *T*_D_) normalized to the integral over the ±1 peaks. (e) Integral *g*^(2)^(0, *T*_D_) monotonically decreases with increasing of the time delay *T*_D_ showing the appearance of a partial photon antibunching in the tail of the PL decay. Excitation wavelength 525 nm, pulse duration 2 ps, pulse repetition rate 100 kHz, power density 5 × 10^–3^ W/cm^2^.

The MAPbI_3_ crystals under study also show pronounced PL blinking when the PL intensity jumps from one intensity level to another (Figure 2(a)). Hence, we can choose photons not only by the time delay *T*_D_ passed after the excitation pulse, but also by the PL intensity level they belong to. Figure 2(b) shows normalized PL decay curves of crystal #7 calculated for the PL photons belonging to the “dim” and “bright” intensity levels marked by different colors in the PL trace in panel (a) (see SI SN4). The PL decays demonstrate similar long tails in the μs region. However, the initial decay time for the dim level is much faster due to the presence of the metastable “supertrap”. This faster initial PL decay correlates with a stronger decrease of *g*^(2)^(0, *T*_D_) for the dim level which reaches ∼0.15 at *T*_D_ ∼ 5.7 μs (Figure 2(d)) in comparison with the lowest level of 0.5 at *T*_D_ ∼ 9.5 μs for the bright level (Figure 2(c)).

**Figure 2:**
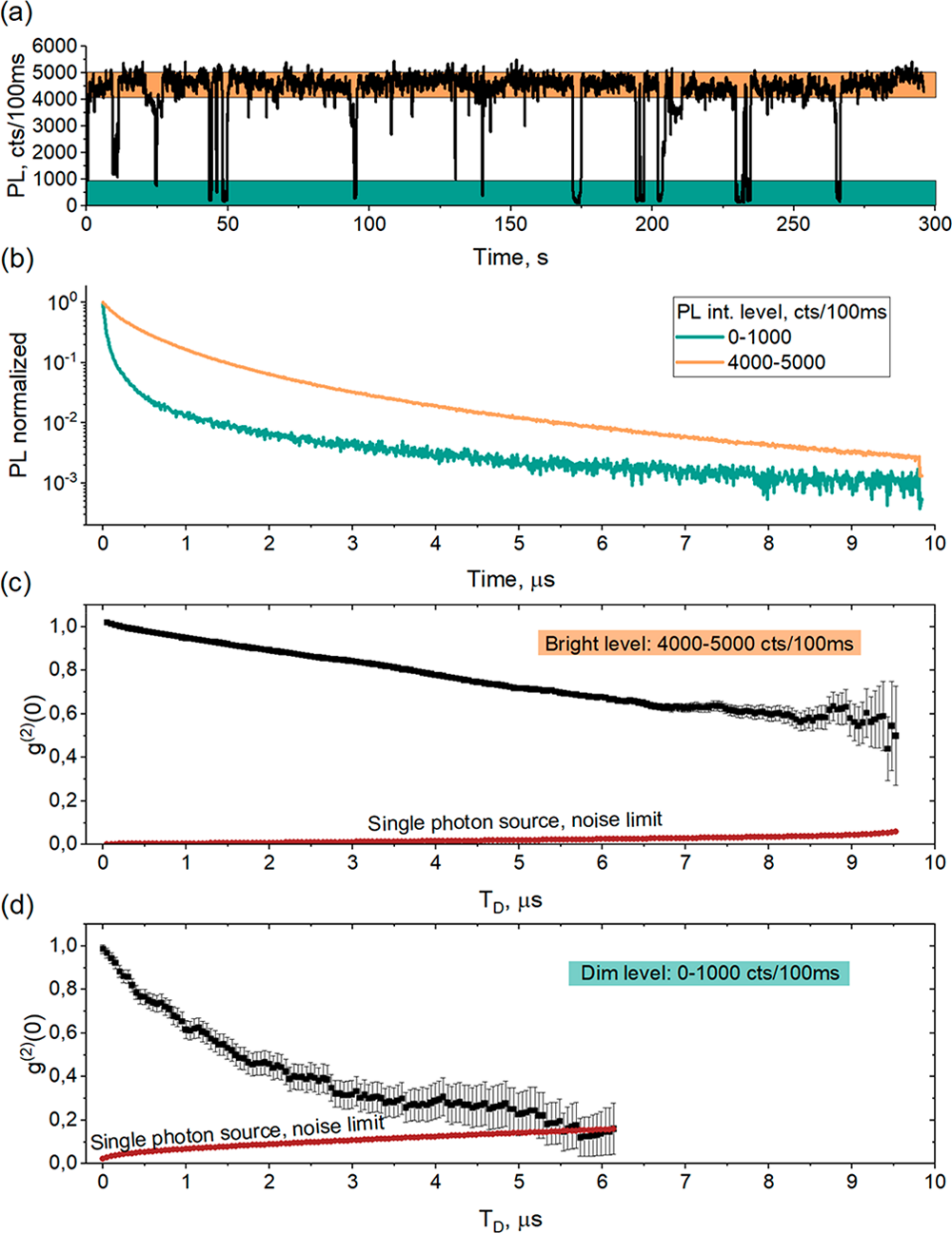
PL intensity transient, PL decay kinetics, and dependence of *g*^(2)^(0) on the time delay *T*_D_ for the bright and dim PL intensity levels for crystal #7. (a) PL intensity trace with bright and dim PL levels marked with orange and green colors, respectively. (b) PL decays calculated for each intensity level marked in (a) with the same color code. (c) and (d) show the dependences of *g*^(2)^(0) (black) as a function of the time delay for the bright and dim intensity levels, respectively. The low limit of *g*^(2)^(0) due to the detector dark counts (dark red line) is shown on both graphs. Note that we are not able to calculate *g*^(2)^(0) for very large *T*_D_ because the error becomes too large due to the small number of photons. That is why *g*^(2)^(0) in (d) does not have any value after 6 μs.

Photon count rates from the PL decay tail in our experiments are very low and often comparable with the noise level of the detector.^40^ It means that even for an ideal single photon source *g*^(2)^(0) cannot reach zero due to the contribution of noise with Poisson statistics. We estimated the lowest value of *g*^(2)^(0) reachable with our detector noise and the particulate count rates for the bright and dim intensity levels of crystal #7 (Figure 2, dark red lines in (c) and (d)), see details in SI SN7A. We can conclude that the photon statistics of the PL tail of this crystal in its dim level reaches that of a single photon source and *g*^(2)^(0, *T*^D^) is not zero (0.15) only because of the noise of our detector. However, the PL of the same crystal in the bright level shows only partial antibunching to which the extent of *g*^(2)^(0, *T*^D^) = 0.5 is not affected by the detector noise. So, this particular perovskite crystal is not a single photon source in delayed PL when its PL is bright, while it becomes a single photon source when its PL is partially quenched.

The effect of partial antibunching in the delayed PL is observed for 22 out of 28 studied crystals (see SI SN8 for several examples). For 6 crystals no indication of antibunching is observed in their pronounced delayed PL as exemplified in Figure 3. Note that the size of this crystal is around one micrometer (Figure 3(d)). At the same time, among the crystal without antibunching there are several which are smaller than 300 nm (diffraction-limited images). We also need to mention that in some cases we observed *g*^(2)^(0) > 1 at early times. One known process leading to photon bunching is fluorescence blinking^13^ which then must be at a microsecond time scale. There could be other reasons related to complex charge dynamics; we see this effect in some of our Monte Carlo simulations (SI SN10B). This effect requires a dedicated investigation beyond the current study.

**Figure 3:**
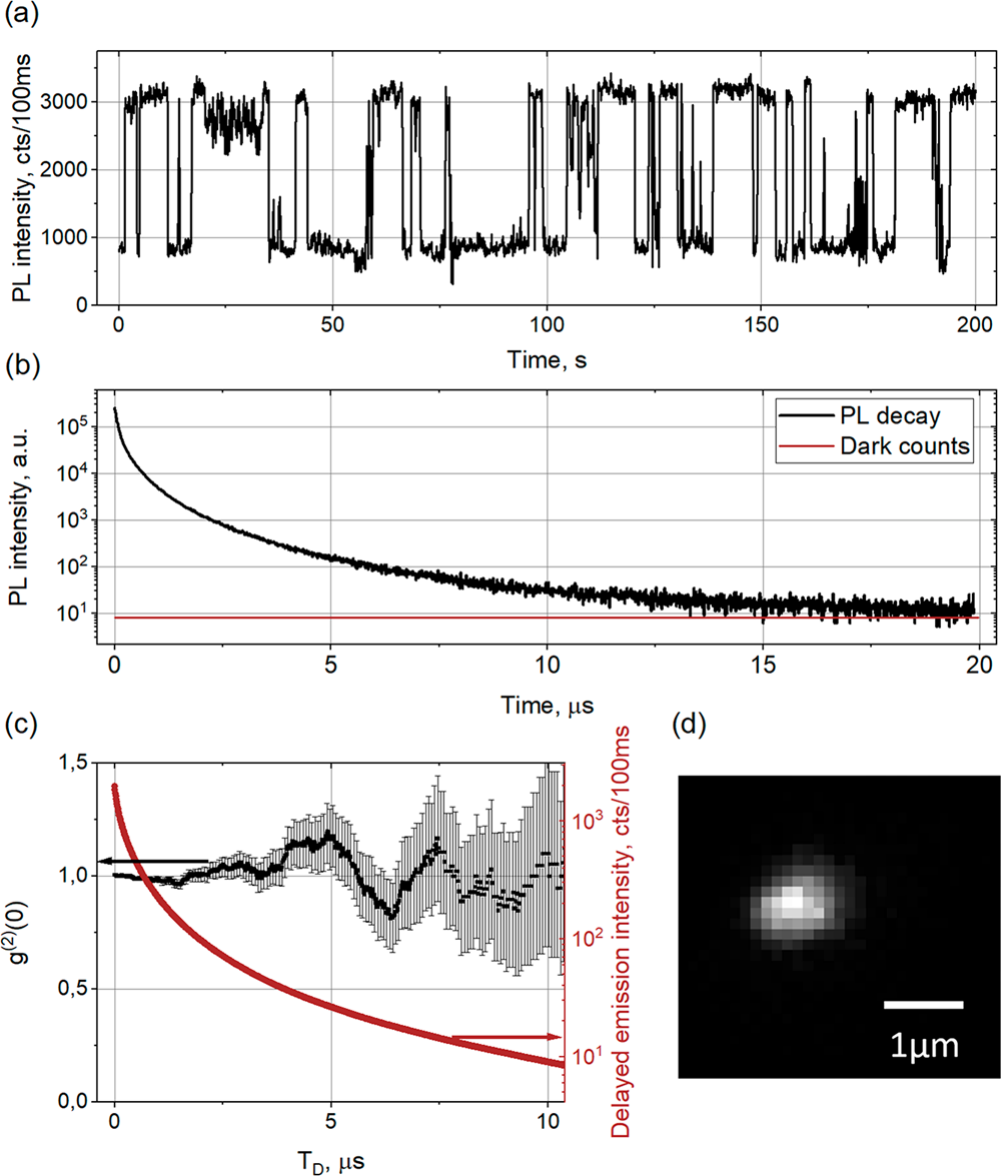
An example of a microcrystal exhibiting PL blinking, but no photon antibunching in the long-living PL. (a) PL trace. (b) PL decay curve (black), dark counts of the detector are also shown by dark red. (c) *g*^(2)^(0, *T*_D_) with no sign of antibunching (black). Delayed PL intensity as function of *T*_D_ is shown by dark red (see SI SN7A). Antibunching is also not found when the photons belonging to the dim intensity level of the PL trace are analyzed separately (not shown). (d) PL image showing that this crystal is larger than the diffraction limit. Excitation wavelength 450 nm, pulse duration 5 ns, pulse repetition rate 50 kHz, power density 5 × 10^–3^ W/cm^2^.

Observation of sub-Poisson statistics (photon antibunching) for the delayed PL component means that there must be a mechanism imposing limitations on the simultaneous emission of two photons from recombination of the long-living electron–hole pairs. One possibility to reach the condition described above for a system initially containing several excitations is to have a cascade of nonlinear recombination processes starting from very fast Auger recombination and bimolecular radiative recombination and finishing with a charge recombination of just one e–h pair at a much longer time scale. This cascade mechanism has been used to explain time-dependent *g*^(2)^(0) for QDs where the initially created short-lived multiexciton state (leading to initially multiphoton emission) quickly disappears leaving just one long-lived exciton state. The latter emits photons with antibunching.^15,16^ We attempt to apply these nonlinear-based mechanisms to our large MAPbI_3_ crystals. Because the initial fast component of the PL decay in the dim level (NC #7) is caused by a fast NR recombination center (supertrap), we need to assume that this NR center works by an Auger mechanism or via an Auger trapping mechanism.^41,42^ Monte Carlo simulations show (see details in SI SN10) that we can fit the PL decay kinetics in the dim state and obtain antibunching for the delayed photons, however, only under the assumption that the nonradiative recombination channel is of the third order which not compatible with trap-assisted Auger recombination (second order). Moreover, the required Auger coefficient (∼10^–25^ cm^6^ s^–1^) is 2–4 orders of magnitude larger than those known for MHPs.^43−45^ Such large coefficients are required simply because we are trying to apply the idea which works in QDs (very small objects) for objects with a volume that is several orders of magnitude larger. So, we think that a cascade of processes starting from an Auger type of charge recombination cannot really explain the effect (see details in SI SN10C).

Figure 4 illustrates our alternative model based on charge detrapping. Let us consider photon statistics of the prompt PL component, which mostly arise from direct recombination of free electrons and holes. The number of radiative recombination events of the prompt PL occurring within the time between two consecutive laser pulses is proportional to the number of absorbed photons during this period. Therefore, it is determined by the Poisson distribution with the average value defined by the number of absorbed photons and the PL quantum yield (PLQY). This consequently leads to absence of the antibunching effect for prompt PL component as has been previously reported for MAPbI_3_.^33^

**Figure 4:**
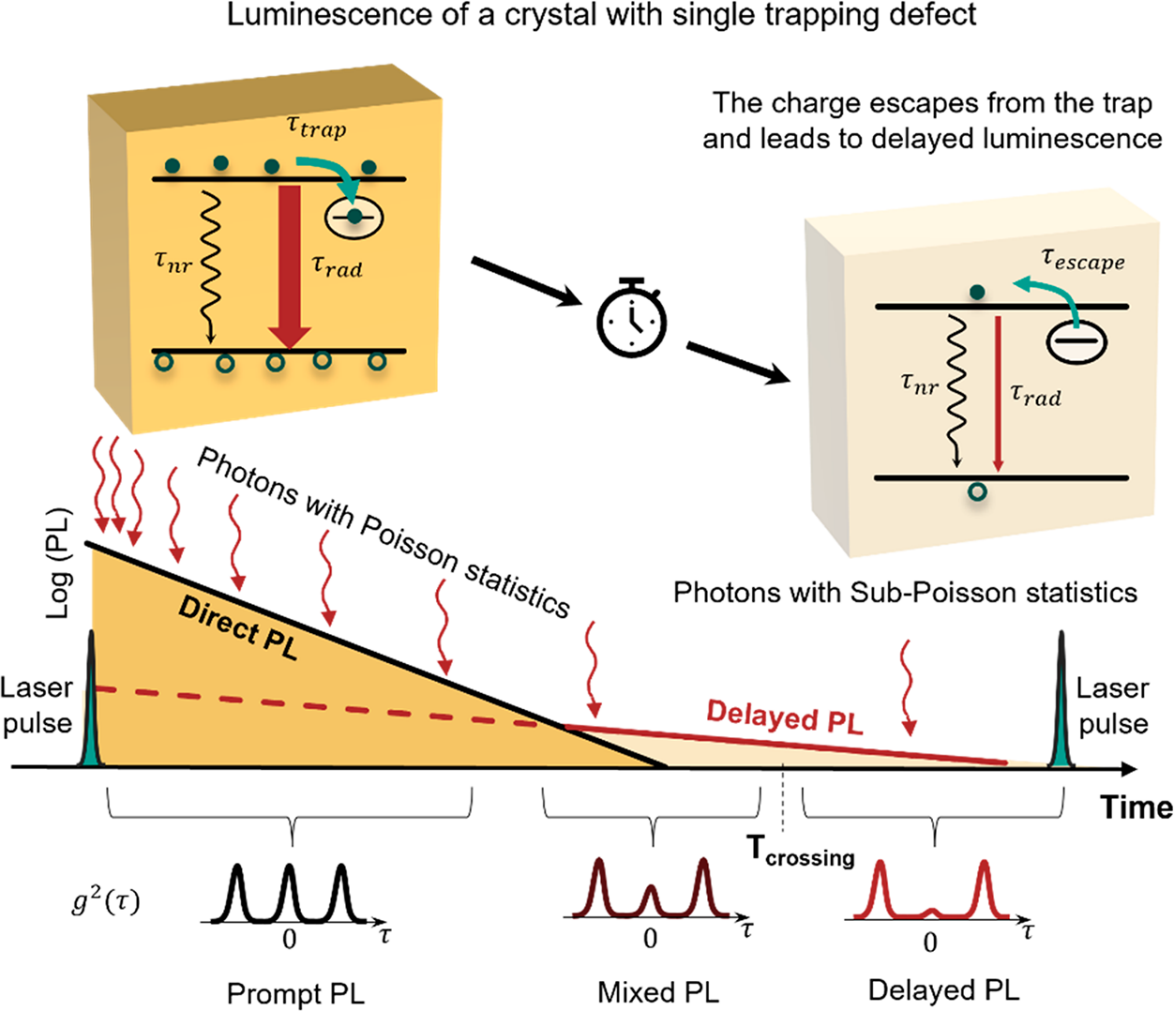
Schematic illustration of the antibunching mechanism in delayed PL of a submicrometer MAPbI_3_ crystal containing one shallow charge trapping defect. Direct PL arises due to radiative recombination of photogenerated e–h pairs. Its intensity is proportional to the number of absorbed and incident photons obeying Poisson statistics. It means that the direct PL should also obey Poisson photon statistics (absence of antibunching). Lifetime of the direct PL is determined by monomolecular recombination due to presence of NR recombination centers. Photons of the delayed PL originate from recombination of charges that are initially trapped and subsequently escaped from the shallow trap. Because there is only one trap in the whole crystal, the delayed PL can be seen as coming from a single emitter (like a single molecule) which cannot emit two photons per excitation pulse because it cannot be double excited (the trap can accept only one charge). In the case of a large difference in the decay times of the direct PL and delayed PL, the photons coming from the tail of the delayed PL should show pure antibunching. *T*_crossing_ is the time when the intensity of the delayed PL starts to dominate over the direct PL.

Since the delayed PL occurs due to the trap-assisted mechanism, the total number of emitted photons is obviously limited by the number of trapping states in the crystal regardless of the excitation light intensity. One trap can accommodate only one electron (or hole). Thermal excitation of this trapped charge carrier back to the band in the microsecond time scale (τ_escape_) and radiative recombination with its counterpart lead to the appearance of one delayed photon. The actual number of photons per one trapped carrier is much smaller than one because it is determined by charge dynamics at the very low charge carrier concentration regime when PLQY is very small.^46^ So, the delayed PL that originated from a single trapping site (or from an outlier in terms of its longer detrapping time in comparison with other trapping sites) should possess perfect photon antibunching *g*^(2)^(0) = 0. If the number of identical trapping sites is *N*, the minimum *g*^(2)^(0) can be estimated by the same equation used for *N* identical independent single photon sources: *g*^(2)^(0) = (*N* – 1)/*N*.^9^

In reality, the PL decay tail contains not only delayed PL due to detrapping, but also a contribution from the direct charge recombination (direct PL) with statistics (Poisson at some conditions) that are different from statistics of a pure single photon emitter. So, the extent of antibunching depends not only on the number of traps, but also on the contribution of the direct PL to the PL decay tail. This contribution can be small if the escape time from the trap (τ_escape_) is much larger than the characteristic decay time of the direct PL (τ_direct_). If we consider a dim state of a blinking crystal (like shown Figure 2 crystal #7), the overall PL decay kinetic is a sum of a fast decay with a large initial amplitude (due to fast monomolecular NR recombination via the supertrap) and a much slower decay with the characteristic time τ_escape_ and a low initial amplitude. Despite the low initial amplitude and possibly very low contribution to the total signal, the delayed PL prevails at times larger than the time which we call here a crossing time delay (*T*_crossing_) as illustrated in Figure 4. From this we can conclude that the larger the difference between τ_escape_ and τ_direct_ and the smaller the initial amplitude of the direct PL vs the delayed PL are, the greater the chance to see photon antibunching in the delayed PL signal.

Let us consider again crystal #7 shown in Figure 2 where *g*^(2)^(0, *T*_D_) is calculated for the bright and dim intensity levels of its PL blinking trace. For the dim level, *g*^(2)^(0) becomes as small as 0.15 for the time delay >5.7 μs (Figure 2(d)) corresponding to the single photon emission limited by the dark noise of our photodetector. In the framework of our model, the long tail of PL in this crystal is caused by only one shallow defect present in the microcrystal (there is only one defect at all, or this defect is an outlier in terms of its longer detrapping time in comparison with other shallow defects). For the bright intensity level, however, only a partial antibunching in the delayed PL is observed. This is because the bright state is not quenched by the supertrap and has a long direct PL decay^30^ leading to a large amount of photons at the long times which screen the emission originated for the trapped charges.^47^ Therefore, shortening of the prompt PL decay due to the presence of the metastable supertrap helps us to infer that this crystal contains only one trapping site that is able to capture a charge carrier for several microseconds. The effect of PL quenching by a supertrap on photon correlations is discussed in detail in SI SN10B.

According to our model, the lowest level of *g*^(2)^(0) depends on the number of the trapping defects which is formally the same as the number of independent single photon sources. At a given defect concentration, the number of defects per crystal is proportional to its volume. Moreover, defect concentration and detrapping times can also be different for different crystals. Therefore, the antibunching effect should vary a lot from crystal to crystal and, in general, be less pronounced for larger crystals. Indeed, we see several crystals without the antibunching effect and the majority of them (e.g., Figure 3) are clearly larger than the diffraction limit of our microscope (∼300 nm).

To check our idea, we used computer simulations to model the PL decays and *g*^(2)^(0, *T*_D_) dependencies for crystal #7 in both bright and dim PL intensity levels (see SI SN10A, 10B for details). We started with modeling of the PL decay in the bright/dim state by solving a Shockley–Read–Hall system of differential equations considering radiative bimolecular recombination, charge trapping and detrapping, and a strong nonradiative recombination channel presented only in the dim state. After that, we used Monte Carlo simulations to calculate *g*^(2)^(0, *T*_D_). In general, we can conclude that our model is able to account for the observations with the recombination constants close to those usually used to model charge recombination in MAPbI_3_.^45^

We estimate sizes of the studied crystals from 30 to 300 nm. Applying this size range to crystal #7 and others where only one shallow trapping defect is present, we obtain the concentration of such defects with several microseconds of detrapping time in the interval 4 × 10^16^ to 4 × 10^13^ cm^–3^. This broad range matches the range of defect concentrations reported for MAPbI_3_ in many studies.^22,27,45^ In the future we are planning to measure sizes of the crystals more precisely by SEM, which will enable accurate estimations of the concentrations of these shallow defect states. Measuring concentrations of defects using effects of antibunching in delayed PL is a quite interesting and rather unexpected application of photon correlation experiments for material science.

To summarize, we discovered photon antibunching in delayed PL of individual MAPbI_3_ perovskite crystals, despite the absence of antibunching in the overall statistics of PL photons. Photon antibunching in the delayed PL is assigned to the presence of a very small number (down to just one) of trapping sites per crystal responsible for the delayed PL. Due to the inability of one trap to capture more than one charge carrier, these large perovskite crystals become sources of delayed single photons. The feasibility of our hypothesis is checked by Monte Carlo simulations. We propose that photon statistics measured and analyzed with time resolution with respect to the excitation pulse allows us to detect very low concentrations of shallow defect states responsible for delayed PL in individual semiconductor particles. This unusual application of photon correlations can become a new ultrasensitive tool for material science and help to rationalize charge dynamics in materials at extremely low excitation conditions.

## Supporting Information Available

Experimental setup and data analysis, sample preparation, additional experimental results, modified Shockley–Read–Hall model considering Auger mechanisms and detrapping, fitting of PL decays at different conditions, and Monte Carlo simulations of *g*^2^(0) (PDF)

## References

[ref1] Carmichael H. J. Photon Antibunching and Squeezing for a Single Atom in a Resonant Cavity. Phys. Rev. Lett. 1985, 55 (25), 2790 10.1103/PhysRevLett.55.2790. 10032239

[ref2] Kimble H. J.; Dagenais M.; Mandel L. Photon Antibunching in Resonance Fluorescence. Phys. Rev. Lett. 1977, 39 (11), 691 10.1103/PhysRevLett.39.691.

[ref3] Basché T.; Moerner W. E.; Orrit M.; Talon H. Photon Antibunching in the Fluorescence of a Single Dye Molecule Trapped in a Solid. Phys. Rev. Lett. 1992, 69 (10), 1516 10.1103/PhysRevLett.69.1516. 10046242

[ref4] Patrick Ambrose W.; Goodwin P. M.; Enderlein J.; Semin D. J.; Martin J. C.; Keller R. A. Fluorescence Photon Antibunching from Single Molecules on a Surface. Chem. Phys. Lett. 1997, 269 (3–4), 365 10.1016/S0009-2614(97)00266-2.

[ref5] Park Y. S.; Guo S.; Makarov N. S.; Klimov V. I. Room Temperature Single-Photon Emission from Individual Perovskite Quantum Dots. ACS Nano 2015, 9 (10), 10386 10.1021/acsnano.5b04584. 26312994

[ref6] Michler P.; Imamoǧlu A.; Mason M. D.; Carson P. J.; Strouse G. F.; Buratto S. K. Quantum Correlation among Photons from a Single Quantum Dot at Room Temperature. Nature 2000, 406 (6799), 968 10.1038/35023100. 10984045

[ref7] Lounis B.; Bechtel H. A.; Gerion D.; Alivisatos P.; Moerner W. E. Photon Antibunching in Single CdSe/ZnS Quantum Dot Fluorescence. Chem. Phys. Lett. 2000, 329 (5–6), 399 10.1016/S0009-2614(00)01042-3.

[ref8] Stangl T.; Wilhelm P.; Remmerssen K.; Höger S.; Vogelsang J.; Lupton J. M. Mesoscopic Quantum Emitters from Deterministic Aggregates of Conjugated Polymers. Proc. Natl. Acad. Sci. U. S. A. 2015, 112 (41), E5560 10.1073/pnas.1512582112. 26417079 PMC4611623

[ref9] Hollars C. W.; Lane S. M.; Huser T. Controlled Non-Classical Photon Emission from Single Conjugated Polymer Molecules. Chem. Phys. Lett. 2003, 370 (3–4), 393 10.1016/S0009-2614(03)00091-5.

[ref10] Wientjes E.; Renger J.; Curto A. G.; Cogdell R.; van Hulst N. F. Strong Antenna-Enhanced Fluorescence of a Single Light-Harvesting Complex Shows Photon Antibunching. Nat. Commun. 2014, 5 (May), 1 10.1038/ncomms5236. PMC4083440 24953833

[ref11] Kumar P.; Lee T. H.; Mehta A.; Sumpter B. G.; Dickson R. M.; Barnes M. D. Photon Antibunching from Oriented Semiconducting Polymer Nanostructures. J. Am. Chem. Soc. 2004, 126 (11), 3376 10.1021/ja031921n. 15025436

[ref12] Hedley G. J.; Schröder T.; Steiner F.; Eder T.; Hofmann F. J.; Bange S.; Laux D.; Höger S.; Tinnefeld P.; Lupton J. M.; Vogelsang J. Picosecond Time-Resolved Photon Antibunching Measures Nanoscale Exciton Motion and the True Number of Chromophores. Nat. Commun. 2021, 12 (1), 1327 10.1038/s41467-021-21474-z. 33637741 PMC7910429

[ref13] Lupton J. M.; Vogelsang J. Photon Correlations Probe the Quantized Nature of Light Emission from Optoelectronic Materials. Appl. Phys. Rev. 2021, 8 (4), 041302 10.1063/5.0059764.

[ref14] Zhao J.; Chen O.; Strasfeld D. B.; Bawendi M. G. Biexciton Quantum Yield Heterogeneities in Single CdSe (CdS) Core (Shell) Nanocrystals and Its Correlation to Exciton Blinking. Nano Lett. 2012, 12 (9), 4477 10.1021/nl3013727. 22871126 PMC3482465

[ref15] Nair G.; Zhao J.; Bawendi M. G. Biexciton Quantum Yield of Single Semiconductor Nanocrystals from Photon Statistics. Nano Lett. 2011, 11 (3), 1136 10.1021/nl104054t. 21288042 PMC3278281

[ref16] Mangum B. D.; Ghosh Y.; Hollingsworth J. A.; Htoon H. Disentangling the Effects of Clustering and Multi-Exciton Emission in Second-Order Photon Correlation Experiments. Opt Express 2013, 21 (6), 7419 10.1364/OE.21.007419. 23546125 PMC3635699

[ref17] Papavassiliou G. C.; Patsis A. P.; Lagouvardos D. J.; Koutselas I. B. Spectroscopic Studies of (C10H21NH3)2PbI4, (CH3NH3)(C10H21NH3)2Pb2I7, (CH3NH3) PbI3, and Similar Compounds. Synth. Met. 1993, 57 (1), 3889 10.1016/0379-6779(93)90530-A.

[ref18] Shamsi J.; Urban A. S.; Imran M.; de Trizio L.; Manna L. Metal Halide Perovskite Nanocrystals: Synthesis, Post-Synthesis Modifications, and Their Optical Properties. Chem. Rev. 2019, 119 (5), 3296 10.1021/acs.chemrev.8b00644. 30758194 PMC6418875

[ref19] Goetz K. P.; Taylor A. D.; Paulus F.; Vaynzof Y. Shining Light on the Photoluminescence Properties of Metal Halide Perovskites. Adv. Funct Mater. 2020, 30 (23), 1910004 10.1002/adfm.201910004.

[ref20] Jena A. K.; Kulkarni A.; Miyasaka T. Halide Perovskite Photovoltaics: Background, Status, and Future Prospects. Chem. Rev. 2019, 119 (5), 3036 10.1021/acs.chemrev.8b00539. 30821144

[ref21] Chen R.; Li J.; Dobrovolsky A.; González-Carrero S.; Gerhard M.; Messing M. E.; Chirvony V.; Pérez-Prieto J.; Scheblykin I. G. Creation and Annihilation of Nonradiative Recombination Centers in Polycrystalline Metal Halide Perovskites by Alternating Electric Field and Light. Adv. Opt Mater. 2020, 8 (4), 1901642 10.1002/adom.201901642.

[ref22] Stranks S. D.; Burlakov V. M.; Leijtens T.; Ball J. M.; Goriely A.; Snaith H. J. Recombination Kinetics in Organic-Inorganic Perovskites: Excitons, Free Charge, and Subgap States. Phys. Rev. Appl. 2014, 2 (3), 034007 10.1103/PhysRevApplied.2.034007.

[ref23] Stranks S. D. Nonradiative Losses in Metal Halide Perovskites. ACS Energy Lett. 2017, 2 (7), 1515 10.1021/acsenergylett.7b00239.

[ref24] Ceratti D. R.; Rakita Y.; Cremonesi L.; Tenne R.; Kalchenko V.; Elbaum M.; Oron D.; Potenza M. A. C.; Hodes G.; Cahen D. Self-Healing Inside APbBr3 Halide Perovskite Crystals. Adv. Mater. 2018, 30 (10), 1706273 10.1002/adma.201706273. 29328524

[ref25] Cahen D.; Kronik L.; Hodes G. Are Defects in Lead-Halide Perovskites Healed, Tolerated, or Both?. ACS Energy Lett. 2021, 6 (11), 4108 10.1021/acsenergylett.1c02027.

[ref26] Scheblykin I. G. Small Number of Defects per Nanostructure Leads to “Digital” Quenching of Photoluminescence: The Case of Metal Halide Perovskites. Adv. Energy Mater. 2020, 10 (46), 2001724 10.1002/aenm.202001724.

[ref27] Jin H.; Debroye E.; Keshavarz M.; Scheblykin I. G.; Roeffaers M. B. J.; Hofkens J.; Steele J. A. It’s a Trap! On the Nature of Localised States and Charge Trapping in Lead Halide Perovskites. Mater. Horiz 2020, 7 (2), 397 10.1039/C9MH00500E.

[ref28] Tian Y.; Merdasa A.; Peter M.; Abdellah M.; Zheng K.; Ponseca C. S.; Pullerits T.; Yartsev A.; Sundström V.; Scheblykin I. G. Giant Photoluminescence Blinking of Perovskite Nanocrystals Reveals Single-Trap Control of Luminescence. Nano Lett. 2015, 15 (3), 1603 10.1021/nl5041397. 25706329

[ref29] Behera T.; Pathoor N.; Phadnis C.; Buragohain S.; Chowdhury A. Spatially Correlated Photoluminescence Blinking and Flickering of Hybrid-Halide Perovskite Micro-Rods. J. Lumin. 2020, 223, 117202 10.1016/j.jlumin.2020.117202.

[ref30] Merdasa A.; Tian Y.; Camacho R.; Dobrovolsky A.; Debroye E.; Unger E. L.; Hofkens J.; Sundström V.; Scheblykin I. G. Supertrap” at Work: Extremely Efficient Nonradiative Recombination Channels in MAPbI 3 Perovskites Revealed by Luminescence Super-Resolution Imaging and Spectroscopy. ACS Nano 2017, 11 (6), 5391 10.1021/acsnano.6b07407. 28485977

[ref31] Bout D. A. V.; Yip W.-T.; Hu D.; Fu D.-K.; Swager T. M.; Barbara P. F. Discrete Intensity Jumps and Intramolecular Electronic Energy Transfer in the Spectroscopy of Single Conjugated Polymer Molecules. Science (1979) 1997, 277 (5329), 1074 10.1126/science.277.5329.1074.

[ref32] Frantsuzov P. A.; Volkán-Kacsó S.; Jankó B. Model of Fluorescence Intermittency of Single Colloidal Semiconductor Quantum Dots Using Multiple Recombination Centers. Phys. Rev. Lett. 2009, 103 (20), 207402 10.1103/PhysRevLett.103.207402. 20366010

[ref33] Eremchev I. Y.; Tarasevich A. O.; Li J.; Naumov A. v.; Scheblykin I. G. Lack of Photon Antibunching Supports Supertrap Model of Photoluminescence Blinking in Perovskite Sub-Micrometer Crystals. Adv. Opt Mater. 2021, 9 (3), 2001596 10.1002/adom.202001596.

[ref34] Chirvony V. S.; Sekerbayev K. S.; Pashaei Adl H.; Suárez I.; Taurbayev Y. T.; Gualdrón-Reyes A. F.; Mora-Seró I.; Martínez-Pastor J. P. Interpretation of the Photoluminescence Decay Kinetics in Metal Halide Perovskite Nanocrystals and Thin Polycrystalline Films. J. Lumin. 2020, 221, 117092 10.1016/j.jlumin.2020.117092.

[ref35] Chirvony V. S.; González-Carrero S.; Suárez I.; Galian R. E.; Sessolo M.; Bolink H. J.; Martínez-Pastor J. P.; Pérez-Prieto J. Delayed Luminescence in Lead Halide Perovskite Nanocrystals. J. Phys. Chem. C 2017, 121 (24), 13381 10.1021/acs.jpcc.7b03771.

[ref36] Becker M. A.; Bernasconi C.; Bodnarchuk M. I.; Raino G.; Kovalenko M. v.; Norris D. J.; Mahrt R. F.; Stoferle T. Unraveling the Origin of the Long Fluorescence Decay Component of Cesium Lead Halide Perovskite Nanocrystals. ACS Nano 2020, 14 (11), 14939 10.1021/acsnano.0c04401. 33174717 PMC7690045

[ref37] Wang Y.; Zhi M.; Chan Y. Delayed Exciton Formation Involving Energetically Shallow Trap States in Colloidal CsPbBr3 Quantum Dots. J. Phys. Chem. C 2017, 121 (51), 28498 10.1021/acs.jpcc.7b09040.

[ref38] Vonk S. J. W.; Fridriksson M. B.; Hinterding S. O. M.; Mangnus M. J. J.; van Swieten T. P.; Grozema F. C.; Rabouw F. T.; van der Stam W. Trapping and Detrapping in Colloidal Perovskite Nanoplatelets: Elucidation and Prevention of Nonradiative Processes through Chemical Treatment. J. Phys. Chem. C 2020, 124 (14), 8047 10.1021/acs.jpcc.0c02287. PMC7217613 32421082

[ref39] Park Y.-S.; Lim J.; Makarov N. S.; Klimov V. I. Effect of Interfacial Alloying versus “Volume Scaling” on Auger Recombination in Compositionally Graded Semiconductor Quantum Dots. Nano Lett. 2017, 17 (9), 5607 10.1021/acs.nanolett.7b02438. 28776995

[ref40] Note that due to a very large luminescence excitation cross-section of the perovskite submicrometer crystals in comparisons with single dye molecules or semiconductor QDs, the contribution of the autofluorescence of the setup to the noise signal is negligible, and that is why we can consider the detector noise only.

[ref41] Abakumov V. N.; Perel V. I.; Yassievich I. N. Nonradiative Recombination in Semiconductors; North-Holland: Amsterdam, 1991.

[ref42] Cohn A. W.; Schimpf A. M.; Gunthardt C. E.; Gamelin D. R. Size-Dependent Trap-Assisted Auger Recombination in Semiconductor Nanocrystals. Nano Lett. 2013, 13 (4), 1810 10.1021/nl400503s. 23464673

[ref43] Shen J.; Zhang X.; Das S.; Kioupakis E.; van de Walle C. G. Unexpectedly Strong Auger Recombination in Halide Perovskites. Adv. Energy Mater. 2018, 8 (30), 1801027 10.1002/aenm.201801027.

[ref44] Herz L. M. Charge-Carrier Dynamics in Organic-Inorganic Metal Halide Perovskites. Annu. Rev. Phys. Chem. 2016, 67 (1), 65 10.1146/annurev-physchem-040215-112222. 26980309

[ref45] Kiligaridis A.; Frantsuzov P. A.; Yangui A.; Seth S.; Li J.; An Q.; Vaynzof Y.; Scheblykin I. G. Are Shockley-Read-Hall and ABC Models Valid for Lead Halide Perovskites?. Nat. Commun. 2021, 12 (1), 3329 10.1038/s41467-021-23275-w. 34099662 PMC8185072

[ref46] Pelant I.; Valenta J. Luminescence Spectroscopy of Semiconductors; Oxford University Press, 2012. 10.1093/acprof:oso/9780199588336.001.0001.

[ref47] For the bright intensity level, *g*^(2)^(0) is still not smaller than 0.5 even for the longest *T*_D_ = 9.5 ns. This means that this delayed PL is a mixture of approximately 30% photons with Poisson statistics and 70% sub-Poisson photons of the delayed PL due to existence of a single trapping defect in the whole crystal (see the calculation procedure in SI).

