# Supplementary material for: Detection of Single Charge Trapping Defects in Semiconductor Particles by Evaluating Photon Antibunching in Delayed Photoluminescence
Source: Nano Lett. 2023 Mar 9;23(6):2087–93. doi: 10.1021/acs.nanolett.2c04004 (PMC10037414; doi:10.1021/acs.nanolett.2c04004)
Supplement: Supplementary file 1 — nl2c04004_si_001.pdf [file nl2c04004_si_001.pdf]

Supporting Information for:

Detection of single charge trapping defects in semiconductor particles by evaluating photon antibunching in delayed photoluminescence

Ivan Yu. Eremchev[1,2,*], Aleksandr O. Tarasevich[1,2,4], Maria A. Kniazeva[1,2,4], Jun Li[3], Andrei V. Naumov[1,2], and Ivan G. Scheblykin[3,*]

[1] Institute of spectroscopy RAS, 108840 Troitsk, Moscow, Russia; [2] Lebedev Physical Institute of the Russian Academy of Sciences, Branch in Troitsk, Moscow, 108840 Russia; [3] Chemical physics and Nano Lund, Lund University, Box 124, SE-22100 Lund, Sweden; [4] National Research University Higher School of Economics, Moscow, 109028 Russia

*E-mail: *eremchev@isan.troitsk.ru; ivan.scheblykin@chemphys.lu.se*

Table of content.

Supplementary Note 1: Experimental setup.

Supplementary Note 2: Samples.

Supplementary Note 3: PL Spectra.

Supplementary Note 4: Calculation of the PL trace, PL decay, $g^{(2)}(\tau)$ function and normalized value of $g^{(2)}(0)$.

Supplementary Note 5: Calculation of $g^{(2)}(\tau, T_D)$ and the integral normalized $g^{(2)}_{norm}(0, T_D)$.

Supplementary Note 6: Calculation of normalized $g^{(2)}(0, T_D)$ function for different PL intensity levels in a flickering PL trace.

Supplementary Note 7: Calculation of the normalized value of $g^{(2)}(0)$ for signal, which is originated from two sources with different photon statistics.

Supplementary Note 7A: Estimation of the delayed photons contribution to the signal as a function of T_D and role of detector noise in low limit of $g^{(2)}_{norm}(0, T_D)$.

Supplementary Note 8: Examples of $g^{(2)}_{norm}(0, T_D)$ obtained for various perovskite nanocrystals with different sizes and blinking dynamics.

Supplementary Note 9: Estimation of number of e-h pairs excited by one laser pulse

Supplementary Note 10: General model of charge recombination, fitting procedure and Monte Carlo simulation of PL decays and $g^{(2)}_{norm}(0, T_D)$.

Supplementary Note 10A: Crystal #7. Fitting of PL decay for the bright intensity with and without charge trapping/de-trapping from shallow traps

Supplementary Note 10B: Crystal #7. Simulation of the PL decay and $g^2(0, T_D)$ for the bright and dim (with supertrap) levels using trapping and de-trapping from shallow traps.

Supplementary Note 10C: Crystal #7. Simulation of the dim level with the supertrap working by a third order Auger mechanism.

References.

Supplementary Note 1: Experimental setup.

Our homebuilt luminescence microscope is able to operate in both the wide-field and scanning confocal modes. The optical magnification is 50 and 100 times for the confocal and wide-field modes, respectively. The luminescence light is collected by an immersion objective lens CarlZeiss 100x, 1.3 NA. The microscope is equipped with a spectrometer to measure luminescence spectra and a photon counting scheme to measure the 2nd order cross-correlation function in the Hanbury Brown and Twiss (HBT) geometry, as well as the absolute arrival times of luminescence single photons.[1,2]

EMCCD camera (Andor Ixon Ultra EMCCD, QE ~ 85 % at 760 nm, dark noise ~ 0.05 counts/pixel/s) is used to record images of single NC in the wide-field mode. The Sol Instruments imaging spectrometer (MS5204i) equipped with a cooled CCD camera (Sol Instruments HS 101H, QE = 80% at 760 nm) is used to measure luminescence spectra (spectral resolution is ~ 0.2 nm for a diffraction grating of 300 grooves/mm).

To measure photon statistics, we use the confocal regime where the luminescence signal is registered in the time-tag mode using the HBT optical scheme consisted of a broadband 50% interference beam splitter (Thorlabs), two identical SPAD-detectors (EG&G SPCM-200PQ, time resolution 1.3 ns, QE ~ 60% at 760 nm, dead time 200 ns, dark counts ~ 25-36 counts/sec) and TCSPC electronics (Becker&Hickl, DPC 230, 165 ps time resolution). The absolute arrival times of PL photons to both detectors are used to calculate second order cross-correlation functions.

The sample was excited either by a picosecond (~ 2 ps) or nanosecond (~ 5 ns) laser sources at wavelengths 525 nm and 450 nm respectively. The first laser source is the stretched second harmonic from the femtosecond laser Tema (Avesta). The second laser is the nanosecond Thorlabs diode laser (NPL45B). The repetition rates of both laser sources are varied in the range from 50 kHz to 500 kHz. The power density of the laser radiation is varied from 5×10^{-4} W/cm^2 to 5×10^{-2} W/cm^2. Most of the experiments were carried out with the laser pulse fluence 0.005 μJ/cm^2. The excitation laser intensity is attenuated by neutral spectral density filters (Standa, Thorlabs). Luminescence signal from $MAPbI_3$ nanocrystals was filtered from the scattered laser radiation by a long-pass filter (Thorlabs FELH 700).

Supplementary Note 2: Samples.

$CH_3NH_3PbI_3$ ($MAPbI_3$, MA^+=$CH_3NH_3^+$) crystals were prepared by the single-step deposition method. 461 mg of PbI_2 and 159 mg of MAI salts were dissolved in 1.25 ml of γ-Butyrolactone (GBL) to obtain the precursor solution. The precursor solution was kept at 60 °C under string for 2 h, and then was further diluted 20 times. To obtain individual $MAPbI_3$ sub-micrometer crystals, 10 μL of dilute solution was spin-cast on a cleaned glass cover slip and annealed at 80°C for 20 minutes in the ambient air environment. The resulting sample was additionally coated for improving stability with a polymer layer (PMMA-Toluene solution, 1 mg/mL) by spin coating of 100 μl at 1460 rpm.

Supplementary Note 3: PL Spectra.

The PL spectra of individual crystals are standard for $MAPbI_3$ films with the peak around 760 nm. Figure S1 shows an example of a $MAPbI_3$ individual nanocrystal spectrum. The Lorentz curve fitting for the PL spectrum is indicated by a gray line. The central wavelengths of the PL peaks from 756 nm to 764 nm for different samples. The excitation wavelength is 450 nm.

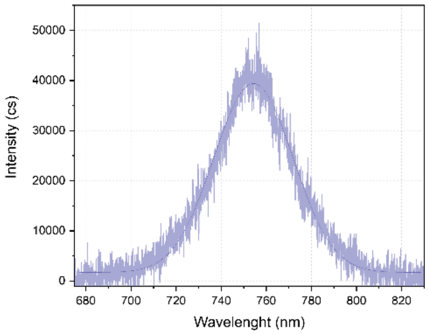

Figure S1. PL spectrum of a single perovskite nanocrystal. The exposure time was 10 s. The power density of the excitation laser was 3.4 ×10^{-1} W/cm^2, pulse repetition rate 100 kHz. Approximation of the spectrum by a Lorentzian function is shown in the figure by the grey line. The luminescence peaks at 756 nm.

Supplementary Note 4: Calculation of the PL trace, PL decay, $g^{(2)}(\tau)$ function and normalized value of $g^{(2)}(0)$.

A home-built software was used to process the time sequences of the arrival of luminescence photons and laser synchro pulses (time-tag data, Figure S2). PL trace is the PL signal temporal evolution $I(t_i)$ with the binning time Δt. It is constructed by summing the number of photocounts N_i detected within sequential time intervals $(t_i,\ t_i + \Delta t)$. Here t_i varies from 0 to the experiment time length T_{exp}, and $t_{i+1} = t_i + \Delta t$.

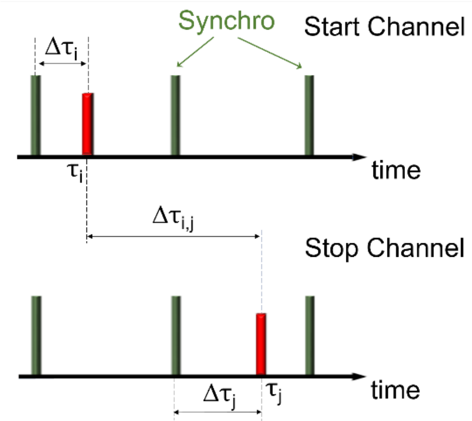

Figure S2. Schematic representation of the time-tag data processing. Time sequences of the arrivals of luminescence photons in the “start” (top) and “stop” (bottom) channels of the HBT scheme and of the laser synchro pulses : τ_i, τ_j - absolute arrival times at the start and stop channels, $\Delta\tau_i, \Delta\tau_j$ - relative arrival times, $\Delta\tau_{i,j}$ - time interval lengths between photon detection events in start and stop channels.

The luminescence decay curves are plotted as histograms of the relative times $\Delta\tau_i, \Delta\tau_j$ (see notations in Figure S2) between the registration events of PL photons by the start and

stop channels and the corresponding laser synchro pulses. To calculate PL decay for a particular PL intensity interval $(I_1; I_2)$ we use the similar procedure but only for the photons, which absolute arrival times τ_i, τ_j correspond to the time t_i of the PL trace and PL intensity $I(t_i)$ belongs to the required interval ($I_1 < I(t_i) < I_2$)

The second order cross-correlation function $g^{(2)}(\tau)$ is calculated as a distribution of the time interval lengths ($\Delta\tau_{i,j} = \tau_i - \tau_j$) between all events of registration photons on the first (τ_i) and second (τ_j) channels of the HBT scheme (see notations in Figure S2). The upper graph in Figure S3 shows an example of amplitude normalized $g^{(2)}(\tau)$ plotted in the time range from $-\frac{3}{2}T_{rep}$ to $\frac{3}{2}T_{rep}$. By the amplitude normalization we mean that $g^{(2)}(\tau)$ is normalized to the average height of the +1 and -1 peaks (this is the usual normalization). The peak near zero delay (0-peak) corresponds to the coincidence cases in the HBT scheme separated by a period smaller than $\pm\frac{1}{2}T_{rep}$. These coincidence cases correspond to the PL photons pairs arising (mainly) due to photoexcitation by the same laser pulses. The peaks centered at $-T_{rep}$ and T_{rep} (±1st peaks) correspond to the coincidence cases separated (in average) by one laser repetition period. These coincidence cases correspond to the PL photons pairs arising (mainly) due to photoexcitation by two subsequent laser pulses. The ratio of the integrals over the 0 peak and the ±1 peaks is the integral normalized value of $g^{(2)}(0)$:

$$g^{(2)}_{norm}(0) = 2\frac{\int_{-\frac{1}{2}T_{rep}}^{\frac{1}{2}T_{rep}} g^{(2)}(\tau)d\tau}{\int_{\frac{1}{2}T_{rep}}^{\frac{3}{2}T_{rep}} g^{(2)}(\tau)d\tau + \int_{-\frac{3}{2}T_{rep}}^{-\frac{1}{2}T_{rep}} g^{(2)}(\tau)d\tau} \quad (S1)$$

Vertical dashed lines in Figure S3 indicate the integration limits for the calculation of the integral normalized $g^{(2)}(0)$. This integral normalized value of $g^{(2)}(0)$ is more appropriate for our study because it gives the probability of the coincidence events for all photons detected within a certain time interval smaller than the distance between two laser pulses. A great advantage of comparing integrals in comparison with maximum values is a much better signal to noise ratio. It is because the number of coincidence events obeys the Poisson statistics (the fluctuation of the count rate has a shot noise nature). In this case increasing of the average signal (total number of coincidences) leads to growing of the signal to noise ratio. It allows us to obtain reliable $g^{(2)}(0)$ values even for relatively rare coincidence events for photons belonging to the PL decay tail.

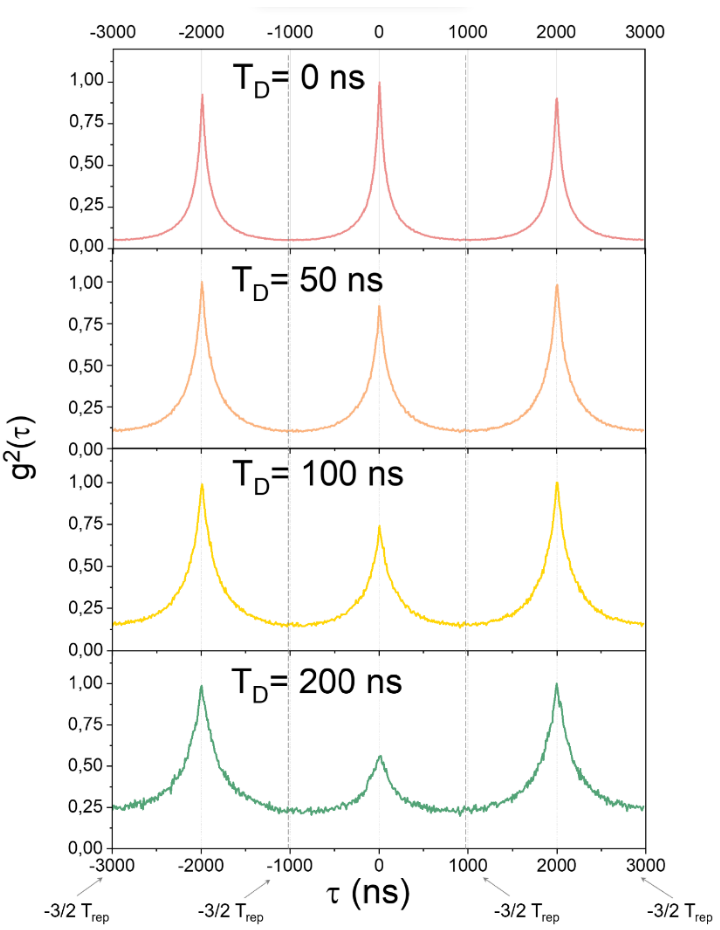

Figure S3. An example of cross-correlation function $g^{(2)}(\tau)$plotted in the time range from $-\frac{3}{2}T_{rep}$ to $\frac{3}{2}T_{rep}$ at different T_D, where T_{rep} is the laser pulse repetition period. Vertical dashed lines show the integration limits the calculation of the normalized value of $g^{(2)}(0)$.

Supplementary Note 5: Calculation of $g^{(2)}(\tau, T_D)$ and the integral normalized $g^{(2)}_{norm}(0, T_D)$.

Like in the previous case, the function $g^{(2)}(\tau, T_D)$ is calculated as a normalized histograms of time intervals ($\Delta\tau_{i,j} = \tau_i - \tau_j$) between detection events on the first (τ_i) and second channels (τ_j) of the HBT scheme (see examples in Figure S3 for $T_D > 0$), however, we consider only delayed PL photons – the photocounts, which are detected with time delay larger than T_D after the corresponding laser excitation pulse: $\Delta\tau_i, \Delta\tau_j \geq T_D$, where $\Delta\tau_i, \Delta\tau_j$ – relative photon detection times. All other photons are ignored. Filtered in this way the delayed photons detection events are schematically shown in Figure S4. In the second time slot of the start channel and in the first time slot of the stop channel of HBT scheme photons are detected before delay time T_D and therefore is ignored in further calculation of $g^{(2)}$-function. Therefore, only one coincidence between start and stop channels $\Delta\tau_{i-1,j}$ gives a contribution to $g^{(2)}$-function.

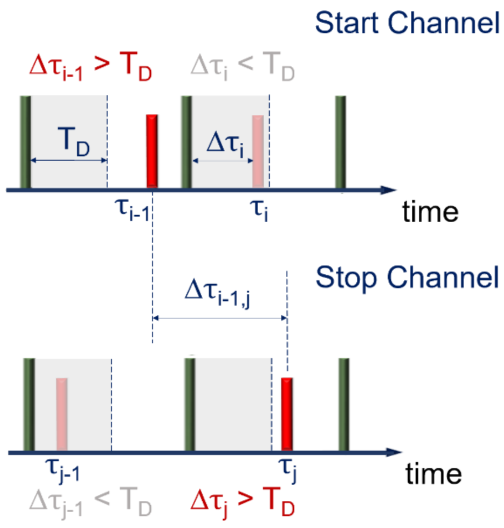

Figure S4. Illustration to the procedure of the time sequence filtering for selecting photons of delayed PL detected with time delay longer than T_D. In the second time slot of the start channel and in the first time slot of the stop channel of HBT scheme photons are detected before the delay time T_D and therefore are ignored in the further calculation of $g^{(2)}$-function. Therefore, only one coincidence between start and stop channels $\Delta\tau_{i-1,j}$ give a contribution to $g^{(2)}$-function. Here we use the same notations like in Figure S2: τ_i, τ_j - absolute arrival times at the start and stop channels, $\Delta\tau_i, \Delta\tau_j$ - relative arrival times, $\Delta\tau_{i,j}$ - time interval lengths between photon detection events in start and stop channels.

The dependence of the integral normalized value $g^{(2)}(0)$ on the delay time T_D is calculated as a ratio of integrals over 0-peak and a half sum of the ±1 peaks of $g^{(2)}(\tau, T_D)$ function (similar to Eq. S1):

$$g_{norm}^{(2)}(0, T_D) = 2\frac{\int_{-\frac{1}{2}T_{rep}}^{\frac{1}{2}T_{rep}} g^{(2)}(\tau, T_D)d\tau}{\int_{\frac{1}{2}T_{rep}}^{\frac{3}{2}T_{rep}} g^{(2)}(\tau, T_D)d\tau + \int_{-\frac{3}{2}T_{rep}}^{-\frac{1}{2}T_{rep}} g^{(2)}(\tau, T_D)d\tau} \qquad (S2)$$

Supplementary Note 6: Calculation of normalized $g^{(2)}(0, T_D)$ function for different PL intensity levels in a flickering PL trace

To calculate ${g_{norm}}^{(2)}(0, T_D)$ for a particular PL intensity interval (I_1;I_2) we use the similar procedure as in Supplementary Note 5 with an additional condition, that only photons, which absolute arrival times τ_i, τ_j correspond to the PL trace time steps t_i with PL intensity $I_1 < I(t_i) < I_2$, are used for calculation. Figure S5 (c,d) show an example of ${g_{norm}}^{(2)}(0, T_D)$ dependencies calculated for the high and low PL intensity levels in PL trace shown in Figure S5 (a).

Supplementary Note 7: Calculation of the normalized value of $g^{(2)}(0)$ for signal, which is originated from two sources with different photon statistics.

The integral normalized value of $g^{(2)}(0)$ for photons, which originates from two independent sources: single emitter with sub-Poisson statistics and a source with Poisson

statistics can be calculate with the help of the expression described in the supplementary information to [1]:

$$g^{(2)}(0) = \frac{\sum_{i=0}^{1}\sum_{j=1}^{i+j\geq 2} p^i P_m^j \left(1-\left(\frac{1}{2}\right)^{i+j-1}\right)}{\left[\sum_{i=0}^{1}\sum_{j=0}^{i+j\geq 1} p^i P_m^j \left(1-\left(\frac{1}{2}\right)^{i+j}\right)\right]^2} \quad (S3)$$

Where $P_m^j = \frac{m^j}{j!}e^{-m}$ – probability to detect j photons per one laser excitation period from the source with Poisson photon statistics, when average number of detected photons is m; p is a probability to detect single photon per one laser excitation pulse from source with Sub-Poisson statistics.

This expression we use to calculate the ratio of PL intensities of sub-Poisson and Poisson sources by a known value of $g^{(2)}(0)$. The first source in our case is associated with the delayed PL due to the single trap, the second is PL resulting from direct charge recombination in a perovskite crystal, which in our case can be described with Poisson statistics.

Moreover, this expression we use to estimate the minimum value of integral normalized $g^{(2)}(0, T_D)$ which should be expected for weak delayed PL signal of an ideal single photon emitter (sub-Poisson statistics) due to influence of detectors dark counts (Poisson statistics), see Supplementary Note 7A.

The average dark count rate is 40 cts/s for our SPADs, let us designate it as I_{dark} . Then the average number of dark counts per one laser repetition period T_{rep} is $m = I_{dark}T_{rep}$. Since the dark counts are uniformly distributed in time, the average number of dark counts per an interval from T_D to T_{rep} of the laser repetition period is $m(T_D) = I_{dark}(T_{rep} - T_D)$. This value is used to calculate P_m^j (probability to detect j photons per laser excitation due to detector dark counts), which is subsequently substituted into the expression for $g_{norm}^{(2)}(0)$.

The average count rate $I(T_D)$ for the delayed photons of PL can be calculated as described in SN4 from the PL decay curve. Then the probability to detect a delayed photon from a single photon emitter with the delay time more than T_D is $p(T_D) = \frac{I(T_D)}{\Delta t}T_{rep}$ per laser excitation pulse, where Δt – binning time, T_{rep} – laser pulse repetition period. This value is substituted into the expression for $g_{norm}^{(2)}(0)$.

We need to mention that the minimum value of integral normalized $g^{(2)}(0)$ is limited due to finite laser pulse duration even for an ideal single photon emitter. Indeed, during the excitation pulse the emitter can be excited more than ones and the probability of such occasion grows as the ratio between the pulse duration and the PL lifetime. However, this does not influence our results because we are interested in the value of $g^{(2)}(0)$ for the delay times exceeding the pulse duration time. Indeed, if we detect a photon coincidence, it means that our emitter firstly was excited, gave a photon and then was excited again. Since the

excitation is possible only during the laser pulse, the first photon must be detected with a delay less than the pulse duration. Therefore, the first photon is rejected since it does not contribute to the delayed PL and does not give a coincidence with the second photon.

When analyzing photons arriving with a large delay after the excitation pulse, we are dealing with very low photon counts rates which can be comparable with the noise level of the detector. Note that due to a very large luminescence excitation cross-section of the perovskite sub-micrometer crystals in comparisons with single dye molecules or semiconductor QDs,[3] the contribution of the autofluorescence of the setup to the noise signal is negligible, that is why we can consider the detector noise only. It means that even for an ideal single photon source the value of $g^{(2)}(0)$ cannot be zero due to the contribution of noise with Poisson statistics.

Supplementary Note 7A: Estimation of the delayed photons contribution to the signal as a function of T_D and role of detector noise in low limit of $g_{norm}^{(2)}(0, T_D)$.

To estimate the contribution of the delayed photons to the total PL signal for the dim and bright PL levels we calculated the delayed PL intensity as a function of T_D. The average delayed emission intensity as a function of delay time T_D (count rate per binning time Δt) is calculated by integration of the PL decay from T_D until the laser pulse repetition period T_{rep} and then normalizing this integral to the total number of time intervals $T_{exp}/\Delta t$ in PL trace. The normalization is required to be able to compare the average delayed emission intensity with the average intensity $I(t_i)$ of where all photons are counted over the time interval $(t_i, t_i + \Delta t)$.

To calculate the average emission intensity in a particular intensity interval $(I_1 ; I_2)$ the similar procedure is used. For that we integrate the PL decay function calculated for the particular PL intensity interval $(I_1 ; I_2)$ and normalize it to the total time PL when the PL intensity belongs to the interval $(I_1 ; I_2)$.

The example of such dependencies for crystal #7 are shown by the red lines in Figure S5 c,d for the bright and dim intensity levels respectively. The scale of the delayed emission intensity is the same in all panels and can be compared. Now we can estimate this lowest limit of $g^{(2)}(0)$ for our experimental data presented in Figure S5. To do so, we assume that the PL photons (with the particular count rates shown in Figure S5 for the dim and bright intensity levels) are coming from single photon sources with intrinsic $g^{(2)}(0)=0$. PL photons are detected with SPADs with the noise level of 40 cts/s per channel equally distributed over the interval between the laser pulses. The expected $g^{(2)}(0, T_D)$ are shown in Figure S5 c,d by yellow lines labeled "Single photon source, noise limit". From Figure S5 c we conclude that the value of $g^{(2)}(0) = 0.15$ at $T_D > 5.7$ µs measured for the dim intensity level fully originates from the SPAD´s dark noise contribution. It means that for the dim PL level the photon statistics in the PL decay tail of crystal #7 corresponds to a pure single photon source. The count rate of this part of delayed PL with $T_D >5.7$ µs is ~ 20 cts/100 ms. For the brighter intensity levels, however, the measured $g^{(2)}(0)$ values are in the range 0.6 - 0.5 for the tail of the decay substantially exceeding the noise limit of $g^{(2)}(0)$ expected for a single photon source. Therefore, the $g^{(2)}(0)$ value in this case are real and not affected by the noise. So, this particular perovskite crystal

is not a single photon source in delayed PL when its PL is bright, while it becomes a single photon source when its PL is partially quenched.

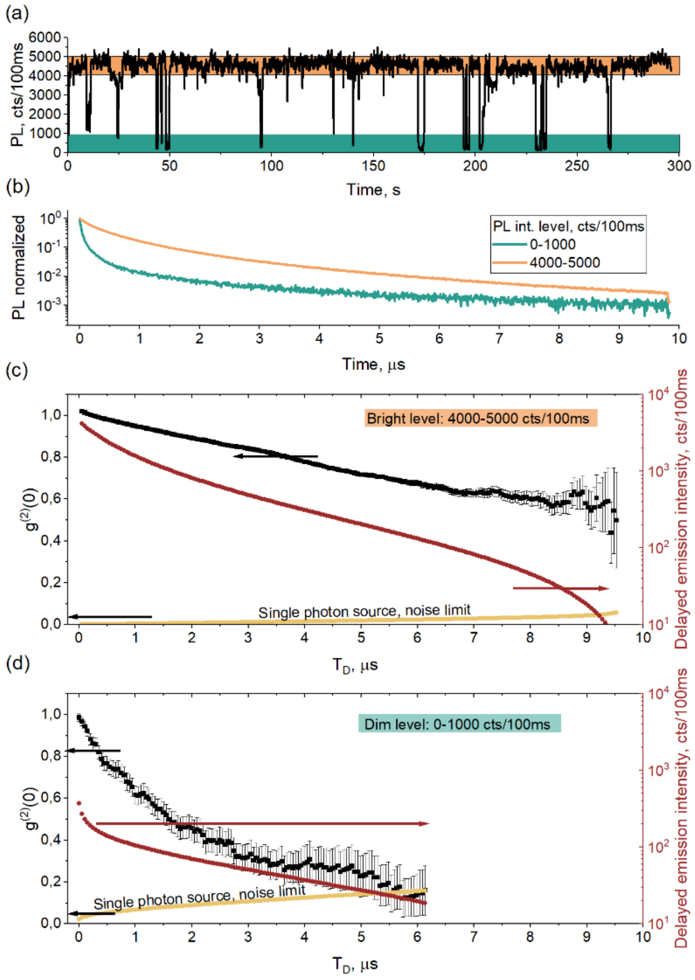

Figure S5. PL intensity transient, PL decay kinetics and dependence of $g^{(2)}(0)$ on the delay time T_D for the bright and dim PL intensity levels for crystal #7. (a) PL intensity trace with bright and dim PL levels marked with orange and green colors respectively. (b) PL decays calculated for each intensity level marked in (a) with the same color code. (c) and (d) show the dependences of $g^{(2)}(0)$ (black) and the integrated intensity of the delayed PL (red) as a function of the delay time for the bright and dim intensity levels, respectively. The low limit of $g^{(2)}(0)$ due to the detector dark counts (dark yellow line) is shown on both graphs. Note that we are not able to calculate $g^{(2)}(0)$ for very large T_D because the error becomes too large due to small number of photons. That is why $g^{(2)}(0)$ in (d) does not have any value after 6 μs.

Supplementary Note 8: Examples of $g^{(2)}_{norm}(0, T_D)$obtained for various perovskite nanocrystals with different sizes and blinking dynamics.

Figures S6-S11 show various examples of $g^{(2)}(0, T_D)$ dependencies for various sizes and morphologies of $MAPbI_3$ nano and micro crystals. The crystal sizes vary from several hundreds of nanometers to a few microns. We examine 28 crystals in total, among them 22 exhibit the photon antibunching phenomenon in the delayed PL and 6 do not. Figures S6-8 demonstrate examples of absence of the antibunching, while Figures S9-11 illustrate a substantial antibunching effect in delayed PL for the selected intensity levels.

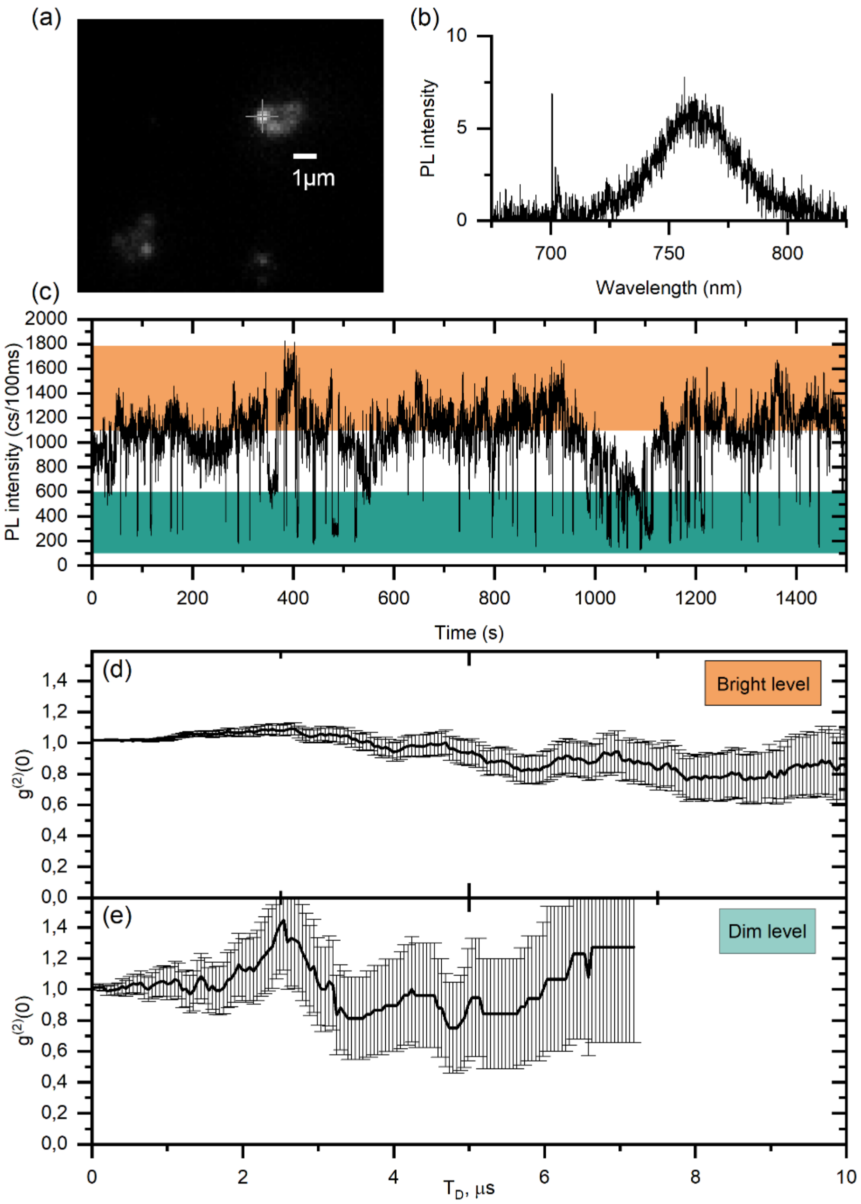

Figure S6. An example illustrating absence of antibunching in delayed PL for a MAPI crystal. Excitation power density – 2.2×10^{-2} W/cm^2, pulse frequency – 50 kHz, wavelength – 450 nm. (a) Luminescence microscopy image of the crystal where PL of the bright spot with sub-diffraction size indicated by the cross (we measured a fragment of a larger crystal) is studied. (b) PL spectrum. The exposure time is 100 s; (c) PL trace. Yellow and pink colors highlight the intensity ranges corresponding to the high and the low levels of PL respectively. (d,e) $g^{(2)}(0, T_D)$ for the high and low PL levels.

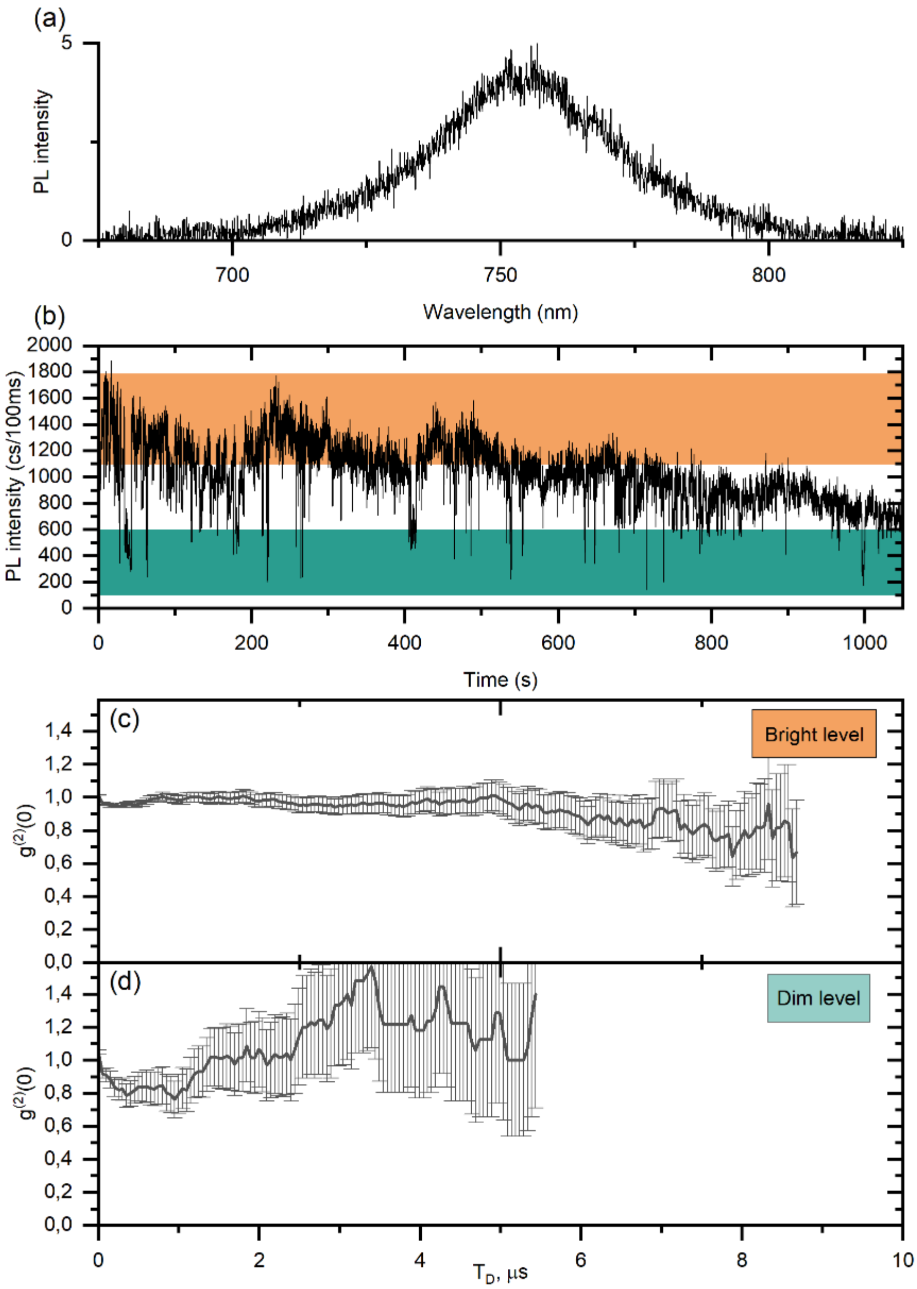

Figure S7. An example illustrating absence of antibunching in delayed PL of a MAPI crystal. The excitation power density - 4.2×10^{-2} W/cm^2, the pulse frequency - 100 kHz, the wavelength – 450 nm. (a) PL spectrum. The exposure time is 10 s; (b) PL trace. Yellow and pink colors highlight the intensity ranges corresponding to the high and the low levels of PL respectively. (c,d) $g^{(2)}(0, T_D)$ for the high and low PL levels.

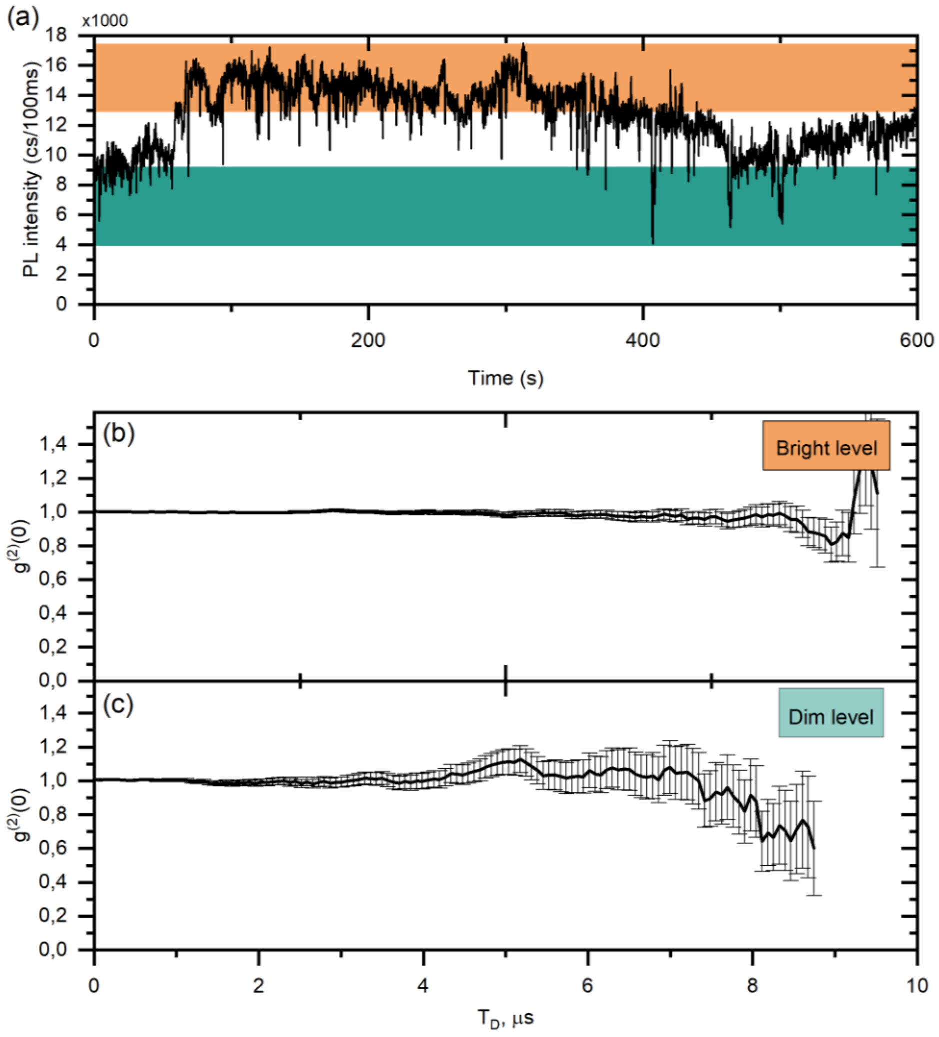

Figure S8. An example illustrating absence of antibunching in delayed PL of a MAPI crystal. Excitation power density - 1.2×10^{-2} W/cm^2, pulse frequency - 100 kHz, wavelength – 450 nm. (a) PL trace. Yellow and pink colors highlight the intensity ranges corresponding to the high and the low levels of PL respectively. (b,c) $g^{(2)}(0,T_D)$ for the high and low PL levels.

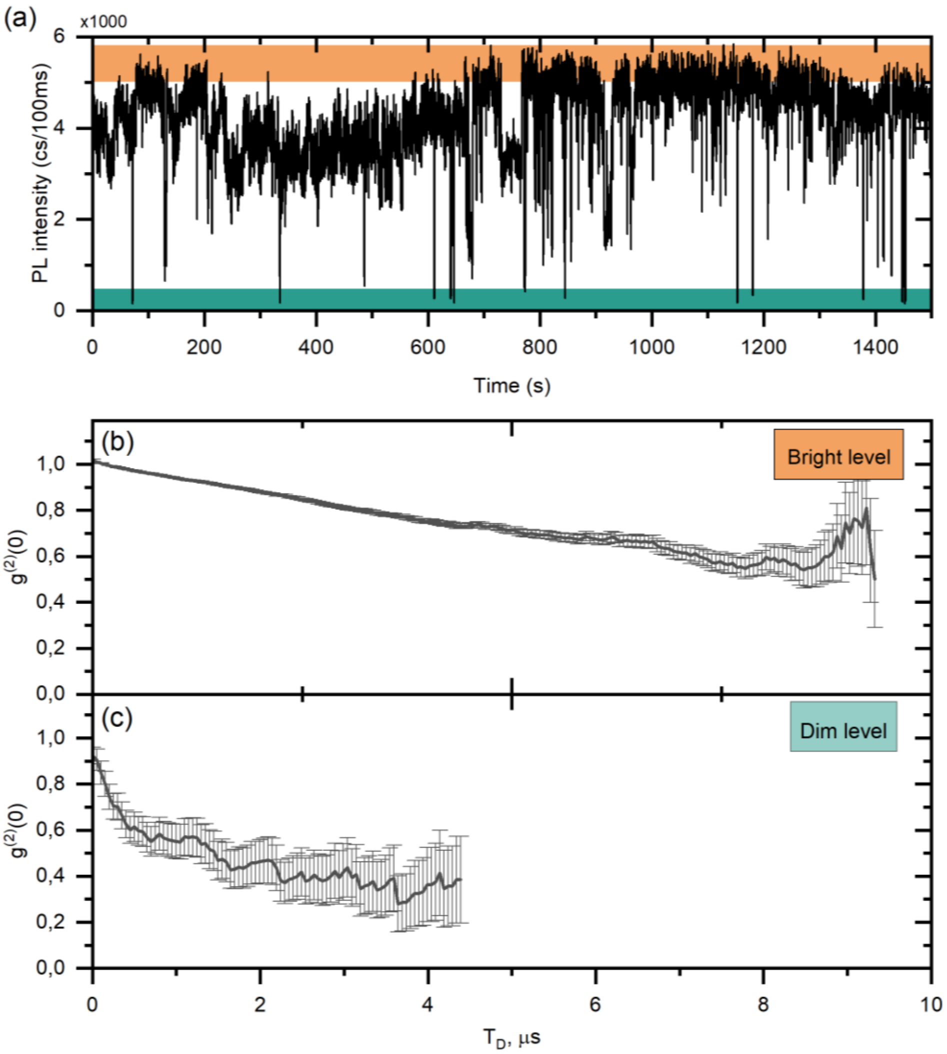

Figure S9. An example illustrating the strong antibunching effect in delayed PL of a $MAPbI_3$ crystal. Excitation power density - 5×10^{-3} W/cm^2, pulse frequency - 100 kHz, wavelength - 525 nm. (a) PL trace. Yellow and pink colors highlight the intensity ranges corresponding to the high and the low levels of PL respectively. (b,c) $g^{(2)}(0,T_D)$ for the high and the low intensity levels, respectively.

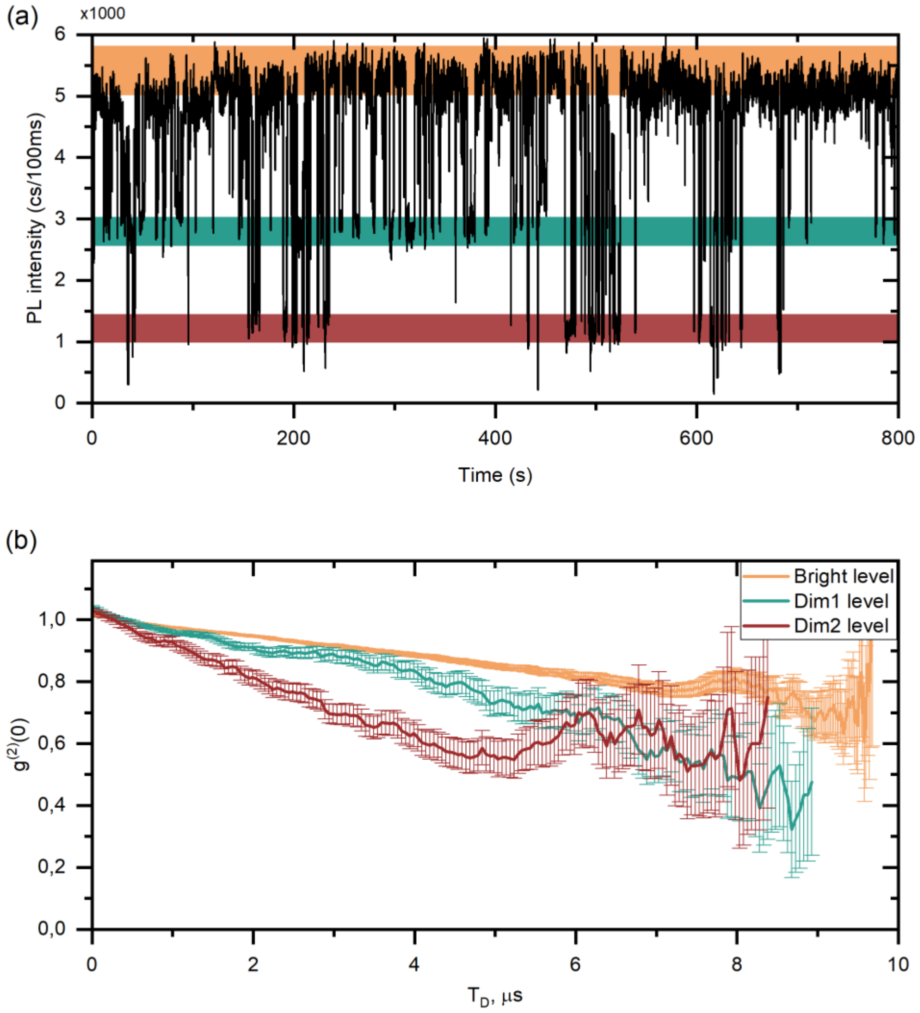

Figure S10. An example illustrating the strong antibunching effect in delayed PL of a MAPI crystal. The power density of the excitation laser is 5×10^{-3} W/cm^2, the laser pulse frequency is 100 kHz. (a) PL trace with blinking; (b) Dependences of $g^{(2)}(0)$ on the luminescence photons delay time for the PL intensity levels marked with different colors.

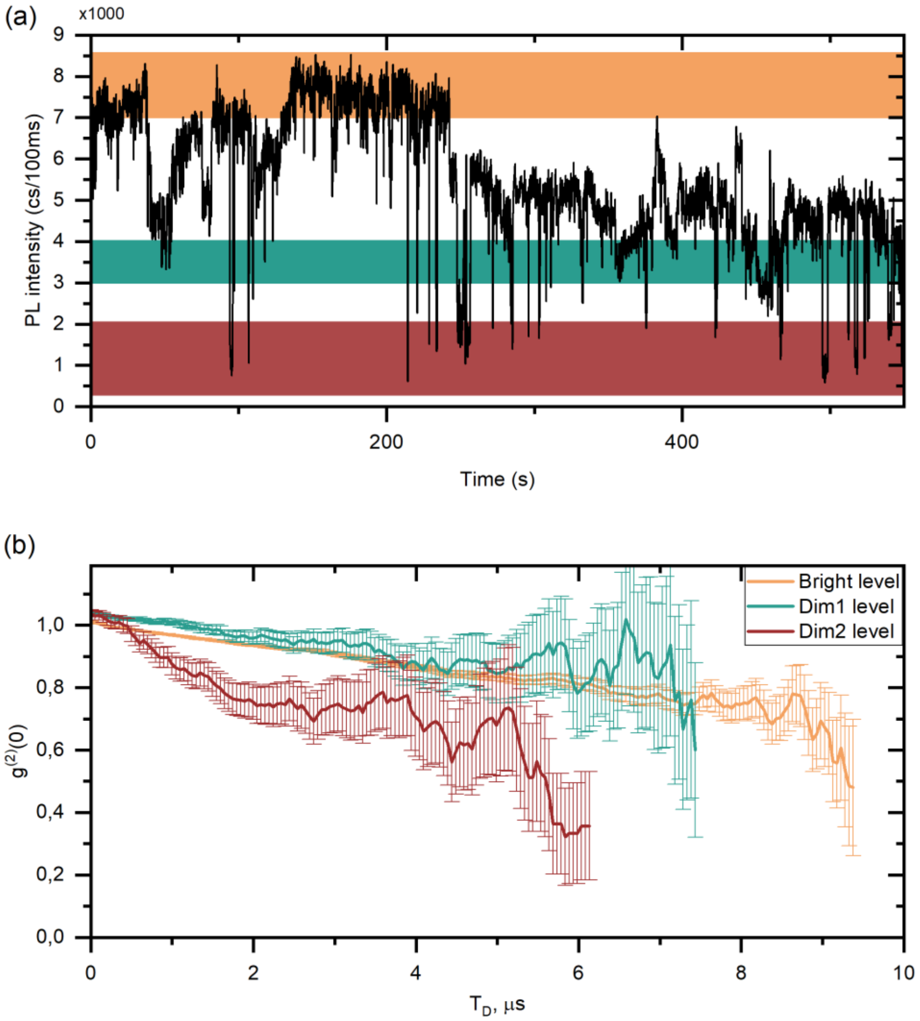

Figure S11. An example illustrating the strong antibunching effect in delayed PL of a MAPI crystal. The power density of the excitation laser is 5×10^{-3} W/cm^2, the laser pulse frequency is 100 kHz. (a) PL trace with blinking; (b) Dependences of $g^{(2)}(0)$ on the luminescence photons delay time for the PL intensity levels marked with different colors.

Supplementary Note 9: Estimation of the number of e-h pairs excited by one laser pulse.

The number of e-h pairs N_{e-h}, excited by one laser pulse is given by:

$$N_{e-h} = P * S * \left(1 - e^{-\frac{d}{l_{abs}}}\right), \quad \text{(S4)}$$

where:

- P is the pulse fluence ($\sim 2 * 10^{11} ph/cm^2$ in our case),
- S is the perovskite crystal cross section,
- d is the perovskite crystal thickness,
- l_{abs} is the absorption length of MAPbI3 (~100nm at 525nm).

The problem here is that we do not know the sizes of our crystals exactly that is why we can only estimate upper and lower limits of the number of e-h pairs based on lower and upper limits of the crystal size.

Upper limit of the average number of e-h pairs. The luminescent image of crystal#7 is diffraction limited. We assume that its size is smaller than 200 nm. It means that the cross section S is smaller than $S_{max} = 4 \times 10^{-10} cm^{-2}$. If we suppose that $d_{max} \leq \sqrt{S_{max}}$ (based on our previous measurements of this samples on atomic force microscope), the upper limit of number of e-h pairs: $N_{e-h} \leq 69$, and the corresponding e-h concentration is $n_{e-h} = 8.6 \times 10^{15} cm^{-3}$ (for a cubic crystal 200x200x200 nm).

Lower limit on average number of e-h pairs. On the other hand, if we know the maximum quantum yield and number of emitted photons per pulse N_{em}, we can find the lower limit of N_{e-h}: $N_{e-h} = N_{em}/ QY$. The maximum quantum yield of MAPbI3 under PMMA film on glass substrate is 40% [4]. The average count rate in bright intensity level for crystal #7 is $CR = 0.45\, ph/pulse$. The photon detection efficiency β in our scheme is 15% (see SI to [1]). Therefore, the lower limit of e-h pairs is $N_{e-h} \geq CR/(\beta * QY_{\max}) = 7.5$, and the corresponding e-h concentration is $n_{e-h} = 1.4 * 10^{16} cm^{-3}$ (for a cubic crystal 82x82x82 nm).

So, for crystal#7 we have the following estimations:

Average number of e-h pairs: $N_{e-h} = 7.5 - 70$.

Concentration of e-h pairs: $n_{e-h} \approx 10^{16} cm^{-3}$.

Supplementary Note 10: General model of charge recombination, fitting procedure and Monte Carlo simulation of PL decays and $g^{(2)}_{norm}(0, T_D)$

To describe the shape of PL decays we used the modified Shockley-Read-Hall model (SRH), that includes different types of recombination channels (Figure S12) characterized by the following rates:

1) radiative rate $\gamma_r = k_r np$, where n and p are concentrations of free electrons and holes in the conduction band and valence bands respectively;
2) trapping rate $\gamma_t = k_t(N - n_t)n$ and Auger trapping rate $\gamma_E = k_E(N - n_t)np$ of electrons. They are followed by a non-radiative recombination of the trapped electrons with holes with the rate $\gamma_n = k_n n_t p$, where N is a concentration of traps and n_t is a concentration of trapped electrons;
3) trapping rate $\gamma_t^{(sh)} = k_t^{(sh)}(N^{(sh)} - n_t^{(sh)})n$ and Auger trapping rate $\gamma_E^{(sh)} = k_E^{(sh)}(N^{(sh)} - n_t^{(sh)})np$ of electrons by shallow traps. This trapping is followed by de-trapping to the conducting band $\gamma_{dt}^{(sh)} = k_{dt}^{(sh)} n_t^{(sh)}$, where $N^{(sh)}$ is a concentration of shallow traps and $n_t^{(sh)}$ is a concentration of electrons trapped by the shallow traps;
4) Auger recombination rate $\gamma_A = k_A np^2$.

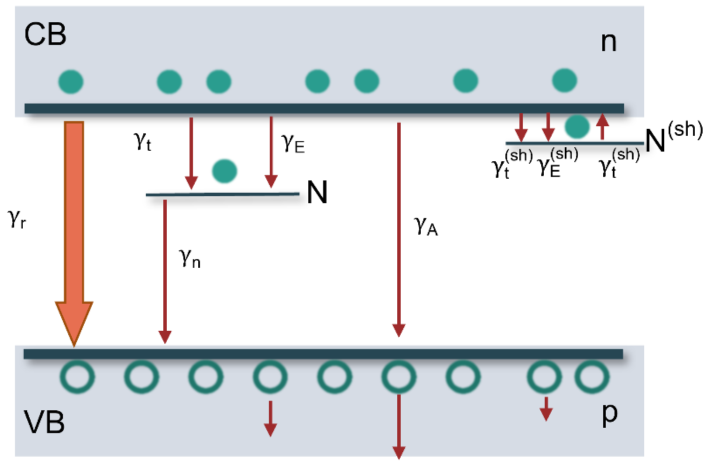

Figure S12. Modified Shockley-Read-Hall model, the corresponding rates are indicated, the text for details.

Combining all these rates together we can write the following system of differential equations:

$$\begin{cases} \dot{n} = -k_r np - \sum\limits_{i:\,traps} \left(k_t^{(i)}(N^{(i)} - n_t^{(i)})n + k_E^{(i)}(N^{(i)} - n_t^{(i)})np\right) - k_a np^2 + k_{dt}^{(sh)} n_t^{(sh)} \\ \dot{n}_t = k_t(N - n_t)n + k_E(N - n_t)np - k_n n_t p \\ \dot{n}_t^{(sh)} = k_t^{(sh)}(N^{(sh)} - n_t^{(sh)})n + k_E^{(sh)}(N^{(sh)} - n_t^{(sh)})np - k_{dt}^{(sh)} n_t^{(sh)} \\ \dot{p} = -k_r np - k_n n_t p - k_A np^2. \end{cases} \quad (S5)$$

Eq.S5 is solved numerically using periodic initial conditions: $n(0) = n_0 + n(T), p(0) = p_0 + p(T), n_t(0) = n_t(T), n_t^{(sh)}(0) = n_t^{(sh)}(T)$, where T is a laser pulse repetition period. The time-dependence of the photoluminescence intensity is then giving by the expression:

$$PL(t) = k_r n(t)p(t). \quad (S6)$$

To compare this solution with our experimental PL decays, the experimental decays are normalized by the number of excitation periods, when the system is in bright (or dim) state. Optimum rate constants $\{k_{r\_opt}, k_{t\_opt}, k_{n\_opt}, \ldots\}$ are found using non-linear least squares method (Levenberg-Marquardt algorithm) to fit the decay with the function Eq.S6.

To investigate the photon statistics for the system described by Eq.S5 we used the Monte-Carlo simulation method. It allows us to simulate the process of charge carrier recombination by calculating the time chain of stochastic transitions, which are radiative and non-radiative recombination, trapping and de-trapping. In every excitation cycle the initial number n_0 (Poisson distributed with average value - N_{e-h}) of electrons and holes is added to the number of electrons n and holes p remained from the previous excitation cycle.

At each simulation time step in every excitation cycle, the probability of different transitions for each electron is calculated as the product of the corresponding rate constant with the *timebin* and the number of possible transitions for this electron which depends on the number of holes in the valence band *p* and the number of unoccupied traps N-n_t. The time step of the simulation ($timebin$=165 ps in our case) is chosen so that the total probability of at least one transition per time step is much less than unity. Whether a particular transition occurs or not during the simulation time interval is then determined by the Monte Carlo method. To simulate the time-tag data like in a real experiments the obtained radiative recombination events are stochastically equally distributed between the two registration channels of HBT scheme. Using this data the functions $g^{(2)}(\tau)\ and\ g^{(2)}_{norm}(0, T_D, I_{PL})$ and the others described in SN4-SN7 are calculated.

Supplementary Note 10A: Crystal #7. Fitting of PL decay for the bright intensity with and without charge trapping/de-trapping from shallow traps

Figure S13 shows examples of the PL decays fits for the bright intensity level for crystal # 7. Figure S13 a presents the best fit (orange) of the experimental PL (blue) using bimolecular recombination radiative channel with rate γ_r and non-radiative monomolecular channel consisted of electron trapping with rate γ_t and recombination of this electron with a hole at rate γ_n (see Supplementary Note 10 for the notations). The PL decay after 2 µs cannot be adequately described by this model. The rate constants are given in the inset of the Figure S13a. The average number n_0 of photoinduced e-h pairs is 10 per excitation laser pulse per crystal volume. Increasing of n_0 leads to even larger discrepancies between the experimental curve and the best approximation.

Figure S13 b shows the best fit (orange) of the experimental PL (blue) by the same model as above where we add electrons trapping by shallow traps (with rate $\gamma_t^{(sh)}$) and their de-trapping with rate $\gamma_{dt}^{(sh)}$. The rate constants are given in the inset of the Figure S13b. In this example two shallow traps in sample volume were considered. One can see that adding the shallow traps allows to fit the PL decay.

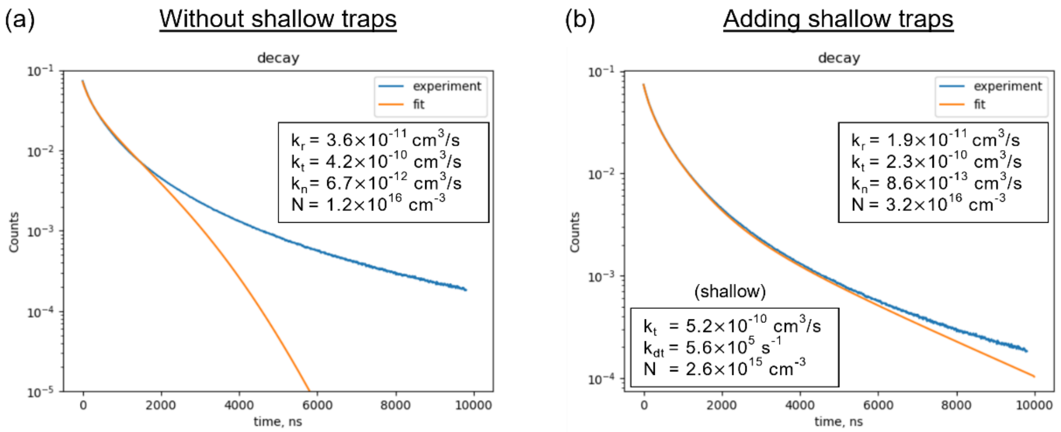

Figure S13. Fit of the PL decay for the bright intensity level using different recombination models. (a) Best fit considering bimolecular recombination radiative channel and non-radiative monomolecular channels (b) Best fit considering bimolecular recombination radiative channel, non-radiative monomolecular recombination channel and trapping-de-trapping by shallow traps.

Supplementary Note 10B: Crystal #7. Simulation of the PL decay and $g^2(0, T_D)$ for the bright and dim (with supertrap) levels using trapping and de-trapping from shallow traps.

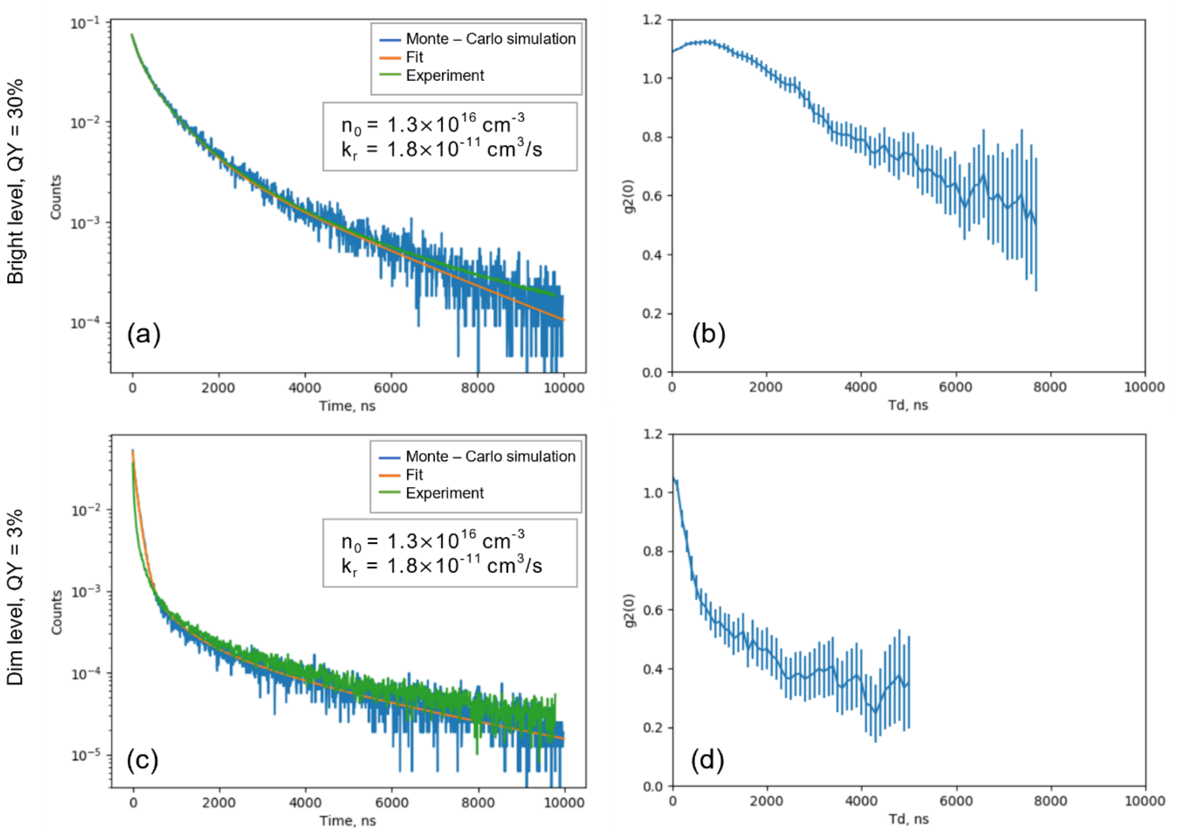

(e)

	NR-traps			Shallow traps		
	k_t, cm^3/s	k_n, cm^3/s	N, cm^{-3}	k_t, cm^3/s	k_n, cm^3/s	N, cm^{-3}
Bright	2.3×10^{-10}	8.5×10^{-13}	3.2×10^{16}	5×10^{-10}	5.6×10^{5}	2.6×10^{15}
Dim	2.3×10^{-10}	8.5×10^{-13}	3.2×10^{16}	1.6×10^{-9}	2×10^{6}	1.3×10^{15}
	Super- trap			3.9×10^{-10}	6×10^{5}	1.3×10^{15}
	4×10^{-9}	5×10^{-9}	1.3×10^{15}	6.2×10^{-10}	4×10^{5}	1.3×10^{15}

Figure S14. Monte-Carlo simulation of the PL decays and $g^2(0, T_D)$ for the bright and dim levels of crystal#7 using trapping/de-trapping from shallow traps. Experimental PL decays (green), PL decay fits (orange), PL decay calculated from Monte Carlo simulation data (blue) for the bright (a) and dim (c) intensity levels. Monte Carlo simulated $g^2(0, T_D)$ for the bright (b) and dim (d) levels. (e) Rate constants and trap concentrations obtained from fittings for the dim and bright levels, which are used for the Monte Carlo simulation. The number of shallow traps per crystal volume: 3 for the dim level, 2 for the bright level. Number of supertraps was 1 for the dim level. Number of non-radiative recombination centers is 25 for both PL intensity levels.

Figure S14 shows examples of the PL decays fits for the bright and dim intensity levels simultaneously for crystal# 7. The fit of PL decay for the bright level is the same as in Figure S13 b. The transition from the bright PL to the dim PL is a result of adding an efficient non-radiative center (supertrap). So, to fit the dim level, we added to the recombination model of

the bright level one effective monomolecular non-radiative recombination channel and also allowed for a slight change in shallow trap parameters. Figure S14 c shows the fit (orange) of experimental PL decay (green) using 3 single shallow traps with different de-trapping times. The rate constants and trap concentrations are given in Figure S14 e. The obtained rate constants and trap concentrations for the dim and bright levels are used for the Monte Carlo simulations of photon emission. The PL decays calculated from Monte Carlo simulated data for the bright and dim levels are shown (blue) in Figure S14 a and c respectively. The simulated $g^2(0, T_D)$ for the bright and dim levels are shown in Figure S14 b and d. Both dependencies $g^2(0, T_D)$ show a clear decrease with time which means appearance of antibunching in the delayed luminescence. The change of $g^2(0, T_D)$ is faster in the dim level.

Figure S15 (orange) shows the PL decay calculated for the dim level with the same parameters as for PL decay in Figure S14 c, but without shallow traps (and, thus, without the de-trapping). The strong exponential decay is due to the monomolecular non-radiative recombination on the supertrap. As one can see, the decay cannot be explained without de-trapping.

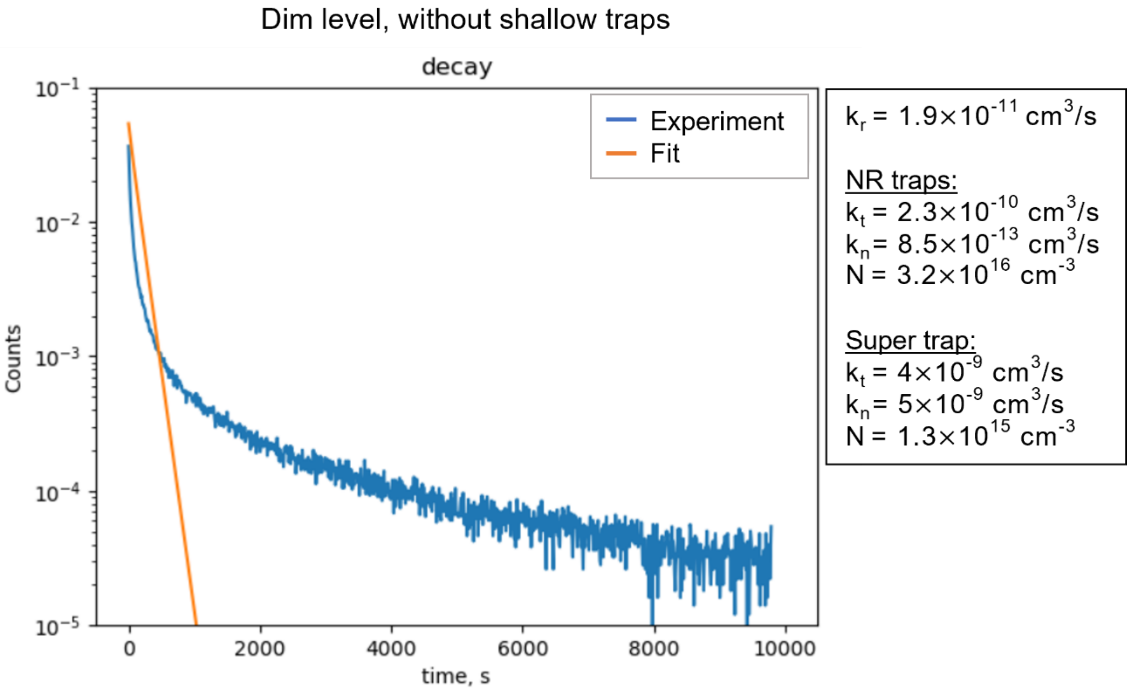

Figure S15. Calculation of the PL decay for the dim level with the same parameters as in Figure S14 but without shallow traps (without de-trapping). The strong exponential decay is due to the supertrap. The decay cannot be explained without de-trapping.

Supplementary Note 10C: Crystal#7. Simulation of the dim level with the supertrap working by a third order Auger mechanism.

Here we present simulations of the dim level of crystal#7 with the supertrap working by a third order Auger mechanism for different numbers of initial excitations. Figure S16 shows experimental PL decays (green), PL decay fits (orange) and PL decay calculated from the Monte Carlo simulation (blue) for the number of initial excitations N_{e-h} = 10, 20, 60. Monte

Carlo simulated $g^2(0, T_D)$ for the same cases are also shown. $g^2(0, T_D)$ decreases with T_D (partial antibunching), also $g^2(0, 0) < 1$ for N_{e-h}= 10, 20. The rate constants and trap concentrations are given for each case in the corresponding panels of the figure.

Although we can model experimental results, the Auger coefficient required for this (10^{-25} cm^6/s) is 2-4 orders of magnitude larger than measured in $MAPbI_3$.[4,5] Moreover, we need to assume that this metastable (single) efficient non-radiative recombination center (supertrap) operates according to the Auger mechanism of the third order instead of the second order which is expected for trap assisted Auger processes.[6]

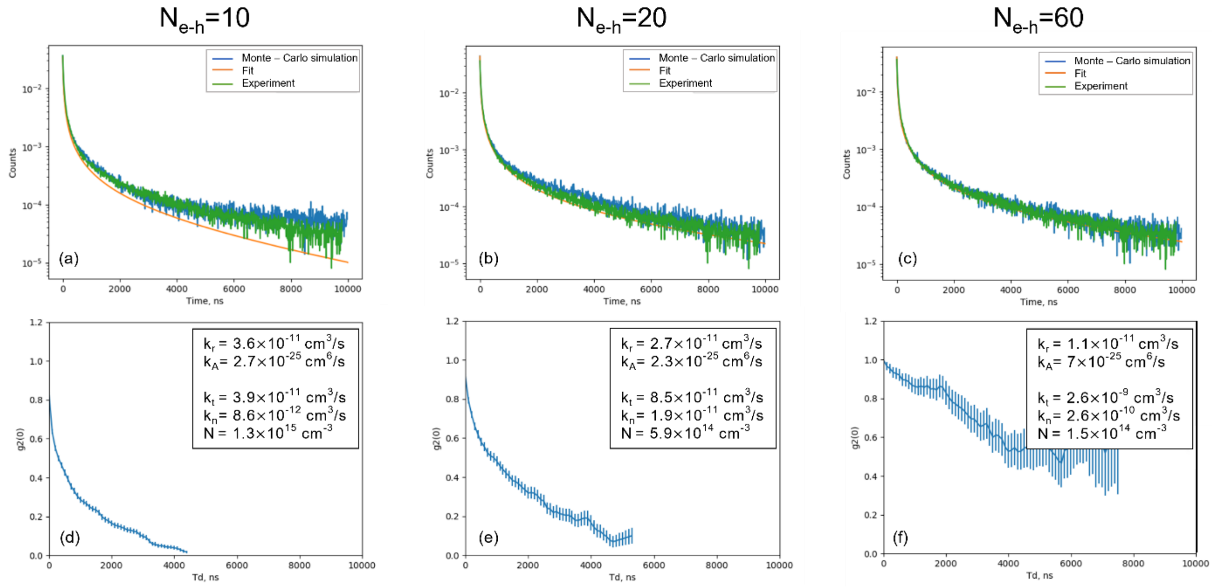

Figure S16. Simulation of the dim level with the supertrap working by a third order Auger mechanism for different numbers of initial excitations. Although we can model our results, the Auger coefficient required for this (10^{-25} cm^6/s) is 2-4 orders of magnitude larger than measured in $MAPbI_3$.

References

(1) Eremchev, I. Y.; Tarasevich, A. O.; Li, J.; Naumov, A. v.; Scheblykin, I. G. Lack of Photon Antibunching Supports Supertrap Model of Photoluminescence Blinking in Perovskite Sub-Micrometer Crystals. *Adv Opt Mater* **2021**, *9* (3), 2001596. https://doi.org/10.1002/adom.202001596.

(2) Eremchev, I. Yu.; Eremchev, M. Yu.; Naumov, A. v. Multifunctional Far-Field Luminescence Nanoscope for Studying Single Molecules and Quantum Dots. *Uspekhi Fizicheskih Nauk* **2019**, *189* (03), 312–322. https://doi.org/10.3367/UFNr.2018.06.038461.

(3) Tian, Y.; Merdasa, A.; Peter, M.; Abdellah, M.; Zheng, K.; Ponseca, C. S.; Pullerits, T.; Yartsev, A.; Sundström, V.; Scheblykin, I. G. Giant Photoluminescence Blinking of Perovskite Nanocrystals Reveals Single-Trap Control of Luminescence. *Nano Lett* **2015**, *15* (3), 1603–1608. https://doi.org/10.1021/nl5041397.

(4) Kiligaridis, A.; Frantsuzov, P. A.; Yangui, A.; Seth, S.; Li, J.; An, Q.; Vaynzof, Y.; Scheblykin, I. G. Are Shockley-Read-Hall and ABC Models Valid for Lead Halide Perovskites? *Nat Commun* **2021**, *12* (1), 3329. https://doi.org/10.1038/s41467-021-23275-w.

(5) Herz, L. M. Charge-Carrier Dynamics in Organic-Inorganic Metal Halide Perovskites. *Annu Rev Phys Chem* **2016**, *67* (1), 65–89. https://doi.org/10.1146/annurev-physchem-040215-112222.

(6) Abakumov, V. N.; Perel, V. I.; Yassievich, I. N. *Nonradiative Recombination in Semiconductors*; North-Holland, Amsterdam, 1991.
